# The glutamate receptor-like 3.3 and 3.6 mediate systemic resistance to insect herbivores in Arabidopsis

**DOI:** 10.1093/jxb/erac399

**Published:** 2022-10-10

**Authors:** Na Xue, Che Zhan, Juan Song, Yong Li, Jingxiong Zhang, Jinfeng Qi, Jianqiang Wu

**Affiliations:** Department of Economic Plants and Biotechnology, Yunnan Key Laboratory for Wild Plant Resources, Kunming Institute of Botany, Chinese Academy of Sciences, Kunming 650201, China; CAS Center for Excellence in Biotic Interactions, University of Chinese Academy of Sciences, Beijing 100049, China; Department of Economic Plants and Biotechnology, Yunnan Key Laboratory for Wild Plant Resources, Kunming Institute of Botany, Chinese Academy of Sciences, Kunming 650201, China; CAS Center for Excellence in Biotic Interactions, University of Chinese Academy of Sciences, Beijing 100049, China; Department of Economic Plants and Biotechnology, Yunnan Key Laboratory for Wild Plant Resources, Kunming Institute of Botany, Chinese Academy of Sciences, Kunming 650201, China; CAS Center for Excellence in Biotic Interactions, University of Chinese Academy of Sciences, Beijing 100049, China; Yunnan Academy of Tobacco Agriculture Science, Kunming 650201, China; Department of Economic Plants and Biotechnology, Yunnan Key Laboratory for Wild Plant Resources, Kunming Institute of Botany, Chinese Academy of Sciences, Kunming 650201, China; CAS Center for Excellence in Biotic Interactions, University of Chinese Academy of Sciences, Beijing 100049, China; Department of Economic Plants and Biotechnology, Yunnan Key Laboratory for Wild Plant Resources, Kunming Institute of Botany, Chinese Academy of Sciences, Kunming 650201, China; CAS Center for Excellence in Biotic Interactions, University of Chinese Academy of Sciences, Beijing 100049, China; Department of Economic Plants and Biotechnology, Yunnan Key Laboratory for Wild Plant Resources, Kunming Institute of Botany, Chinese Academy of Sciences, Kunming 650201, China; CAS Center for Excellence in Biotic Interactions, University of Chinese Academy of Sciences, Beijing 100049, China; James Hutton Institute, UK

**Keywords:** Arabidopsis, glucosinolate, glutamate receptor-like genes, jasmonic acid, metabolome, *Myzus persicae*, *Spodoptera litura*, transcriptome

## Abstract

Herbivory activates responses in local and systemic leaves, and the glutamate receptor-like genes *GLR3.3* and *GLR3.6* are critical in leaf-to-leaf systemic signalling. However, whether and how these genes mediate plant systemic resistance to insects remain largely unexplored. We show that a piercing-sucking insect *Myzus persicae* (green peach aphid, GPA) or chewing insect *Spodoptera litura* (cotton leafworm, CLW) feeding-induced systemic defences were attenuated in the *glr3.3 glr3.6* mutants. In response to herbivory from either insect, *glr3.3 glr3.6* mutants exhibited reduced accumulation of the hormone jasmonic acid (JA) and defensive metabolites glucosinolates (GSs) in systemic (but not local) leaves. Transcriptome analysis indicated that GLR3.3 and GLR3.6 play an important role in regulating the transcriptional responses to GPA and simulated CLW feeding in both local and systemic leaves, including JA- and GS-related genes. Metabolome analysis also revealed that in response to GPA or simulated CLW feeding, GLR3.3 and GLR3.6 are involved in the regulation of various metabolites locally and systemically, including amino acids, carbohydrates, and organic acids. Taken together, this study provides new insights into the function of GLR3.3 and GLR3.6 in mediating transcripts and metabolites in local and systemic leaves under insect attack, and highlights their role in regulating insect resistance in systemic leaves.

## Teaser

GLR3.3 and GLR3.6 are essential for insect feeding-induced systemic defence by regulating transcripts and metabolites under insect attack.

## Introduction

In nature, plants are challenged by various abiotic and biotic stresses, and one of the biotic stresses that plants suffer from is insect feeding. Depending on the insect feeding mode, insects are generally divided into two distinct groups: those with piercing-sucking mouthparts and those with chewing mouthparts. Piercing-sucking insects, such as aphids and whiteflies, insert their slender stylets between cells to establish a feeding site in the phloem without inflicting obvious physical damages to plant tissue, but still cause substantial physiological changes in plants (Howe and Jander, 2008; Louis and Shah, 2013). Chewing insects, such as grasshoppers (Orthoptera), beetles (Coleoptera), and caterpillars (Lepidoptera), cause mechanical injuries to plant tissues during feeding (Howe and Jander, 2008). Interestingly, certain components of oral secretions (OS) of chewing insects may diffuse into the disrupted tissue during feeding and can be perceived by plants, inducing strong defence-related responses (Wu and Baldwin, 2010; Erb and Reymond, 2019).

Defence responses in plants are mediated by an interconnected network of signalling pathways, including phytohormones. Jasmonic acid (JA), a fatty acid-derived plant hormone, has been shown to play a major role in regulating defences against phloem- and chewing-feeding insects in Arabidopsis (Howe and Jander, 2008). In addition to JA, salicylic acid (SA), abscisic acid (ABA), and indole-3-acetic acid (IAA) are also implicated in plant resistance to aphids or caterpillars (Stotz *et al.*, 2002; Bodenhausen and Reymond, 2007; Erb *et al.*, 2012). Perception of herbivory and activation of signalling pathways finally lead to biosynthesis and accumulation of defensive metabolites, which are toxic, deterrent, and/or anti-digestive for insects (War *et al.*, 2012). Glucosinolates (GSs) are a group of defensive secondary metabolites that are characteristic of the Brassicaceae. There are two major chemical groups of GSs, indole and aliphatic GSs, depending on the amino acids that they are synthesized from (Wittstock and Halkier, 2002). Increased levels of both indole and aliphatic GSs reduced herbivory by *Spodoptera exigua* on Arabidopsis (Gigolashvili *et al*., 2007a, b). Compared with aliphatic GSs, indole GSs are more defensive against aphids (Kim *et al.*, 2008).

Insect herbivory not only activates defences in the attacked (local) leaves, but highly elevated defences are mounted in distal intact (systemic) leaves (Erb and Reymond, 2019). The feeding insects often move to other parts of the plants to continue feeding, and insects of the same or different species may concurrently or subsequently attack the systemic leaves or roots, and under these circumstances, systemic defences offer immediate resistance to the insects. It has been identified that the reactive oxygen species (ROS), electrical signals, and cytosolic Ca^2+^ ([Ca^2+^]_cyt_) signals function in long-distance signalling, conveying information from local to systemic leaves in Arabidopsis response to herbivore attack (Choi *et al.*, 2016; Gandhi *et al.*, 2021), and these three signals were found to interact with each other (Maffei *et al.*, 2004; Miller *et al.*, 2009; Zimmermann *et al.*, 2009; Suzuki *et al.*, 2013).

In plants, the rapid transmission of Ca^2+^ signal is dependent on the glutamate receptor-like proteins GLR3.3 and GLR3.6 (Nguyen *et al.*, 2018; Toyota *et al.*, 2018). Vincent *et al.* (2017) used the GCaMP3 fluorescent protein-based [Ca^2+^]_cyt_ sensor and showed that [Ca^2+^]_cyt_ increases at the green peach aphid (GPA) feeding site in detached Arabidopsis leaves, but the aphid-induced [Ca^2+^]_cyt_ elevation was abolished in the *glr3.3 glr3.6* mutant. Furthermore, *Pieris rapae* feeding and mechanical wounding on Arabidopsis expressing GCaMP3 also caused rapid increase of [Ca^2+^]_cyt_, and the Ca^2+^ signals could propagate to distal leaves through plasmodesmata and phloem; the Ca^2+^ signals were similar in the local leaves of wild-type (WT), *glr3.3*, *glr3.6*, and *glr3.3 glr3.6* plants, but were reduced in the systemic leaves of *glr3.3* and *glr3.6* mutants compared with the WT plants, and diminished in the *glr3.3 glr3.6* double mutants (Toyota *et al.*, 2018). Thus, GLR3.3 and GLR3.6 are required for inducing local [Ca^2+^]_cyt_ elevation around the aphid feeding sites and both local and systemic Ca^2+^ signalling upon caterpillar feeding and wounding. In addition to affecting Ca^2+^ signal transmission, GLR3.3 and GLR3.6 have also been reported to function in leaf-to-leaf electrical and ROS signalling upon wounding (Mousavi *et al.*, 2013; Nguyen *et al.*, 2018; Fichman and Mittler, 2021). However, precisely how GLR3.3 and GLR3.6 are linked to plant resistance to herbivores remains largely unknown. Although the *glr3.3 glr3.6* mutants were susceptible to *Spodoptera littoralis* and the *Myzus persicae* fecundity was not altered on *glr3.3 glr3.6* mutants (Vincent *et al.*, 2017; Nguyen *et al.*, 2018),whether GLR3.3 and GLR3.6 function in systemic resistance to herbivores is unknown.

Here, we show that in Arabidopsis, loss of functions of *GLR3.3* and *GLR3.6* led to reduced systemic resistance to both the piercing-sucking GPA and chewing *Spodoptera litura* (cotton leafworm, CLW); consistently, the contents of JA and GSs, as well as the expression of JA and GS biosynthesis-related genes in the systemic leaves of *glr3.3 glr3.6* double mutants were also affected. By integrating transcriptome and metabolome profiling, this study reveals that a series of transcripts and metabolites are regulated by GLR3.3 and GLR3.6. These data provide comprehensive new insights into the role of GLR3.3 and GLR3.6 in plant systemic resistance to insect herbivores.

## Materials and methods

### Plant materials and growth conditions

WT and mutant *Arabidopsis thaliana* (Col-0) seeds were vernalized for 3 d in darkness at 4 °C and germinated on petri dishes containing half-strength Murashige Skoog medium. Seven-day-old seedlings were transferred to individual pots and kept in a chamber at 22 °C under short-day conditions (10 h light/14 h dark, 70 μM photons m^−2^ s^−1^), and after 3 weeks, the plants were used for fluorescence observation; after 6 weeks, the plants were used for GPA or simulated CLW feeding treatment or bioassays. *glr3.3* (salk_099757) and *glr3.6* (salk_091801) mutants were ordered from ABRC (http://arabidopsis.org) and crossed to obtain *glr3.3 glr3.6* double mutants. The eighth (L8) and 13th leaves (L13) of plants were chosen as local and systemic leaves, respectively; counting began with the oldest leaves, which were small and spatulate, and the first leaves (L1) were the oldest on the mature rosette (Farmer *et al.*, 2013).

### DNA cloning and transformation

For construction of the p35S:GCaMP6s Ca^2+^ biosensor, the pGP-CMV-GCaMP6s (plasmid #40753) was purchased from Addgene (USA). The *GCaMP6s* fragment with restriction enzyme sites *Bgl*II and *Bst*EII was inserted into the pCAMBIA3301 vector (Cambia, Canberra, Australia), under the control of a CaMV35S promoter (p35S). The construct was introduced into *Agrobacterium tumefaciens* strain GV3101, which was used to transform WT Arabidopsis and *glr3.3 glr3.6* mutants using the floral dipping method (Clough and Bent, 1998).

### Insect rearing and plant treatment

The GPA colony was originally established from a single nymph collected from a field in the Kunming Institute of Botany, Chinese Academy of Sciences, in Kunming, China, in 2015 (Zhuang *et al.*, 2018). Arabidopsis (Col-0) plants were provided as the food for the GPAs. CLW eggs were supplied by Keyun Biocontrol (China). The eggs were kept in a growth chamber (16 h of light at 26 °C and 8 h of dark at 22 °C, 70 μM photons m^−2^ s^−1^) until the larvae hatched.

For GPA infestation, in the treatment group, 50 adult GPAs were gently placed onto each local L8 of WT plants, *glr3.3* and *glr3.6* single mutants, and *glr3.3 glr3.6* double mutants using a soft brush, and they were enclosed in a clip cage; the plants of the control group were fixed with empty cages. After 24 h and 3 d of GPA feeding, local L8 and systemic L13 from the control and GPA treatment group were collected; leaves from three individual replicated plants were pooled as one replicate and all the samples were immediately frozen in liquid nitrogen. Samples were stored at –80 °C until further use.

For collecting CLW OS, CLW larvae (third to fifth instar), which were reared on Arabidopsis, were gently poked with a pipette to stimulate regurgitation, and the OS were collected on ice with the pipette; the collected OS were immediately divided into small aliquots before being stored at –20 °C. To simulate CLW herbivory, L8 were wounded with a pattern wheel three times on each side of the midvein, to generate six rolls of wounds, and 5 μl (unless indicated otherwise) of *S. litura* OS was gently rubbed onto the fresh puncture wounds (W+OS treatment); the plants of the control group were non-treated. After 2 h and 2 d of W+OS treatment, local and systemic leaves of WT, *glr3.3*, *glr3.6*, and *glr3.3 glr3.6* plants from the control and W+OS treatment group were collected, and leaves from three individual replicated plants were immediately pooled to form one replicate, and all the samples were immediately frozen in liquid nitrogen. Samples were stored at –80 °C until further use.

### Fluorescence microscopy

Arabidopsis plants stably expressing GCaMP6s were imaged with a motorized fluorescence stereo microscope (SMZ-18, Nikon, Japan) equipped with a 13.5× objective lens (NA = 0.156, P2-SHR PLAN APO, Nikon). The GFP-based GCaMP6s was excited using a 440/470 nm excitation filter. The green fluorescent signal passing through a 535/550 nm filter was acquired every 1 s with a sCMOS camera (Photometrics, Canada) using the NIS-Elements imaging software (Nikon).

To mimic *Spodoptera litura* feeding responses, the Arabidopsis leaf 1 was wounded with tweezers, and 2.5 μl of *S. litura* OS was gently rubbed into the fresh puncture wounds. Images were captured for 5 min. To investigate the Ca^2+^ signals induced by GPA feeding, 50 adult GPAs were allowed to infested leaf 3 of each genotype. Images were captured for 13 min after aphid infestation. Each leaf represented one biological replicate. Images were exported as MP4 files for analysis. All images were captured with a constant exposure time (800 ms).

Using the NIS-Elements (Nikon, Japan) imaging software, GCaMP6s signals were analysed over time at regions of interest of systemic leaves after W+OS treatment and GPA feeding. In order to calculate the fractional fluorescence changes (ΔF/F), the equation ΔF/F = (F−F_0_)/F_0_ was used, where F_0_ denotes the average baseline fluorescence determined by the average of F over the first 10 frames of the recording (Keinath *et al.*, 2015; Vincent *et al.*, 2017) before treatment.

### Aphid fecundity assay

At the end of GPA treatment on the local leaves of WT, *glr3.3*, *glr3.6*, and *glr3.3 glr3.6* plants, all aphids were removed. One healthy adult GPA was placed on the systemic leaves (WT, *glr3.3* and *glr3.6* single mutants, and *glr3.3 glr3.6*) of each plant and was enclosed in a clip cage, and the numbers of offspring were counted every day for 14 d.

### CLW growth assay

The local leaves of WT, *glr3.3*, *glr3.6*, and *glr3.3 glr3.6* plants were treated with simulated CLW herbivory (W+OS) or left untreated (control group); after 2 d, freshly hatched *S. litura* neonates were placed on the systemic leaves (WT, *glr3.3* and *glr3.6* single mutants, and *glr3.3 glr3.6*), and the masses were measured 1 d later.

### Quantification of phytohormones and glucosinolates

Phytohormone determination was performed on an HPLC-MS/MS (LCMS-8040, Shimadzu, Japan) according to a method described previously (Song *et al.*, 2022). Contents of glucosinolates in Arabidopsis were analysed by HPLC (LC-20 AD, Shimadzu) following previously described methods (Burow *et al.*, 2006; Qi *et al.*, 2018). Five biological replicates were analysed.

### RNA-seq data acquisition and analysis

Three biological replicates were used for RNA library constructions and sequencing. For RNA-seq data acquisition and analysis, total RNA of leaves was extracted using TRIzol reagent (ThermoFisher Scientific, USA). For RNA-seq data acquisition, Illumina TruSeq RNA Sample Prep Kit (Illumina) was used to construct cDNA libraries. The generated cDNA libraries were sequenced on a NovaSeq 6000 platform (Illumina) to acquire sequence reads (6 G depth). 

The Bioconductor DEseq2 (v3.9) package (Love *et al.*, 2014) was employed to infer differential gene expression. Transcripts with adjusted *P* values ≤0.05 (Wald test) and absolute values of log_2_ (fold change) ≥1 were selected as differentially expressed genes (DEGs). DEGs were obtained by comparing the RNA-seq data of treatment group with the RNA-seq data of the respective control group from the given genotype’s given leaf (local or systemic). Venn diagram analysis and graphs were generated on Venny2.1 (https://bioinfogp.cnb.csic.es/tools/venny/index.html), and KEGG analysis was performed using the OmicShare tools (https://www.omicshare.com/tools).

### Metabolome sample preparation

The freshly frozen Arabidopsis leaves were ground into uniform powder in liquid nitrogen using a tissue grinder (Shanghai Jingxin Industrial Development Co., Ltd., China) and stored at –80 °C. The quality control (QC) sample was generated by pooling equal amounts of all acquired samples. An extraction solution (370 μl of methanol, 450 μl of methyl tert-butyl ether, and 680 μl water) was added to each leaf sample (~100 mg each). The samples were vortexed for 1 min and centrifuged at 18 900 ×*g* for 10 min before the supernatants were transferred to microfuge tubes.

For GC-MS analysis, the substratum (400 μl) was mixed with 100 μl of 4-hydroxybenzoic acid (10 μg ml^–1^ in water) as an internal standard in a 1.5 ml microfuge tube, and dried in a vacuum concentrator (Eppendorf). Then 100 μl of methoxyamine (20 mg ml^–1^ in pyridine) solution was added to each residue and kept at 37 °C for 90 min to perform the oximation reactions for protecting carbonyl and decreasing the number of isomers. Silylation was then performed by adding 80 μl of MSTFA (N-methyl-N-(trimethylsilyl) trifluoroacetamide) to the sample and the reaction was kept for 30 min at 37 °C. The samples were then centrifuged for 5 min at 12 000 ×*g* and the supernatants were transferred to conical inserts of 2 ml glass vials for subsequent GC-MS analysis. For LCMS analysis, each substratum (400 μl) was mixed with 100 μl of triphenylphosphate (10 µg ml^–1^ in methanol) as an internal standard in a 1.5 ml tube, and dried in a vacuum concentrator (Eppendorf). The residues were dissolved with 100 μl of the methanol-water mixture (1:4 v/v). The dissolved solution was centrifuged for 5 min at 12 000 × *g* and the supernatants were transferred to conical inserts of 2 ml glass vials for subsequent LC-MS analysis.

### Metabolome data acquisition and analysis

The sample analyses and separation of metabolites were performed according to a method described previously (Li *et al.*, 2015). Mass calibration was performed using the Bruker Data Analysis software. The calibrated raw data were processed using the MS-Dial 4.6 (Tsugawa *et al.*, 2020). After the process of noise removal, molecular feature extraction, peak alignment, and features annotation, a peak table with quantitative and qualitative information of the detected metabolites was acquired. The FCs (fold changes) of metabolites were obtained by comparing their relative concentrations in the treatment and control group for a given leaf position and given genotype. The differentially produced metabolites (DPMs) were selected if QC-RSD (SD/Average) ≤30% and |log_2_ (FC) |≥1. The DPMs of *glr3.3 glr3.6* vs WT were obtained by calculating the FCs of metabolites between the *glr3.3 glr3.6* and WT plants. The metabolites whose |WT log_2_FC—*glr* log_2_FC| ≥1 and QC-RSD ≤30% were considered to be differentially regulated metabolites (DRMs). The software MetaboAnalyst 5.0 (Pang *et al.*, 2021) was used for pathway analysis, statistical comparison, and visualization.

### Statistical analysis

The relative gene expression (VST) levels and metabolite contents were transformed by log_2_ and normalized by Z-score (Cheadle *et al.*, 2003); Z-score = (xi-μ)/σ, where xi is the VST value of a gene, μ is the mean VST value for the gene across all tissues, and σ is the standard deviation across all tissues. TBtools was employed to construct the heatmaps of transcriptome and metabolome (Chen *et al.*, 2020). Statistical analysis was done using R (https://www.r-project.org). 

## Results

### GLR3.3 and GLR3.6 are required for *Myzus persicae* infestation-induced activation of systemic [Ca^2+^]_cyt_

GLR3.3 and GLR3.6 are essential for both local and systemic Ca^2+^ signalling in response to mechanical wounding and feeding by the lepidopteran insect *Pieris rapae* (Toyota *et al.*, 2018). GPA feeding on Arabidopsis also led to elevated Ca^2+^ signals at the feeding site (Vincent *et al.*, 2017). However, whether GPA induces systemic Ca^2+^ signalling, and if GLR3.3 and GLR3.6 are needed for the systemic Ca^2+^ signalling, are unknown. To visualize Ca^2+^ signals, the GCaMP6s Ca^2+^ sensor was chosen as the cytosolic Ca^2+^ ([Ca^2+^]_cyt_) indicator, which is more sensitive than the GCaMP1 to GCaMP5 sensors (Chen *et al.*, 2013). We transformed the construct harbouring *GCaMP6s* driven by the CaMV35S promoter into WT (Col-0) Arabidopsis and the *glr3.3 glr3.6* double mutant, generating the 35S:GCaMP6s*/*Col-0 and 35S:GCaMP6s*/glr3.3 glr3.6* plants.

**Fig. 1.**
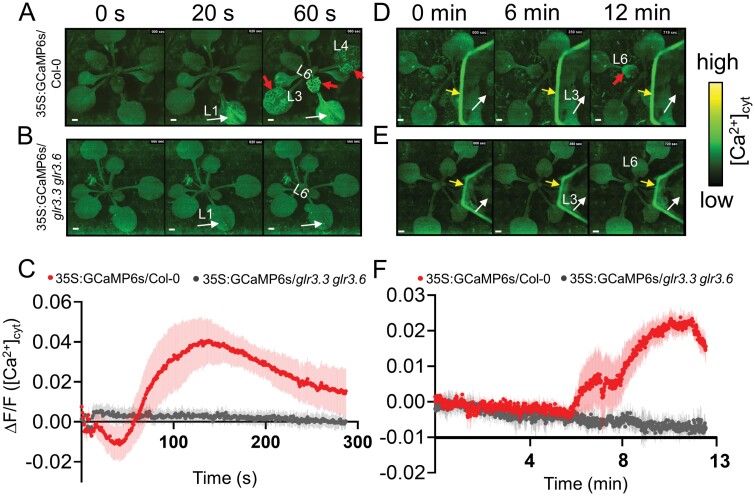
W+OS treatment and GPA infestation induces elevation of [Ca^2+^]_cyt_ in systemic leaves of 35S:GCaMP6s/Col-0 plants but not *35S:GCaMP6s/glr3.3 glr3.6* plants. (A, B) Simulated *Spodoptera litura* feeding (W+OS)-induced [Ca^2+^]_cyt_ signals in (A) 35S:GCaMP6s/Col-0 and (B) *35S:GCaMP6s/glr3.3 glr3.6* plants. White arrows indicate leaf 1 (L1) treated with W+OS, and red arrows indicate systemic leaves (L3, L4, L6), in which [Ca^2+^]_cyt_ increased. (C) Normalized fluorescence (ΔF/F) of the systemic leaves 6 of 35S:GCaMP6s/Col-0 and *35S:GCaMP6s/glr3.3 glr3.6* plants until 300 s after W+OS treatment. (D, E) GPA infestation-induced [Ca^2+^]_cyt_ signals in (D) 35S:GCaMP6s/Col-0 and (E) *35S:GCaMP6s/glr3.3 glr3.6* plants. White arrows indicate the leaf 3 (L3), on which 50 GPAs were infested, red arrows indicate the systemic leaf 6 (L6) whose [Ca^2+^]_cyt_ increased. Yellow arrows indicate the cardboard used to restrict the movement of aphids. (F) Normalized fluorescence (ΔF/F) of the systemic leaves 6 of 35S:GCaMP6s/Col-0 and *35S:GCaMP6s/glr3.3 glr3.6* plants until 13 min after infestation of GPAs. Error bars represent SE of the means; *n*=5. Scale bars =1 mm.

Consistent with a previous study (Toyota *et al.*, 2018), application of *Spodoptera litura* OS to artificial wounds (W+OS) on the 35S:GCaMP6s/Col-0 leaf 1 led to rapid propagation of fluorescent signals around the feeding sites, which subsequently spread to the distal unwounded third, fourth, and sixth leaves within 1 min (Fig. 1A, C; Supplementary Video S1); however, no visible signals were detected from the 35S:GCaMP6s*/glr3.3 glr3.6* plants (Fig. 1B, C; Supplementary Video S2). Furthermore, infestation by 50 GPAs on 35S:GCaMP6s/Col-0 leaf 3 increased the signals of [Ca^2+^]_cyt_, and a clear increase of [Ca^2+^]_cyt_ signal was also observed in the systemic sixth leaf within 13 min (Fig. 1D, F; Supplementary Video S3), although it was weaker than that induced by W+OS. In contrast, GPA infestation-induced signal of [Ca^2+^]_cyt_ was abolished in the 35S:GCaMP6s*/glr3.3 glr3.6* plants (Fig. 1E, F; Supplementary Video S4). Notably, the movement of GPAs also triggered a weak [Ca^2+^]_cyt_ signal, due to the legs touching the Arabidopsis leaves. Thus, the systemic [Ca^2+^]_cyt_ signals resulted from both GPA feeding and movement on local leaves. We inferred that GPA infestation induces local and systemic increase of [Ca^2+^]_cyt_ signals in a GLR3.3- and GLR3.6-dependent manner.

### GLR3.3 and GLR3.6 are required for systemic resistance to *Myzus persicae* and *Spodoptera litura*

The functions of GLR3.3 and GLR3.6 in Arabidopsis defence against *Spodoptera littoralis* and *Myzus persicae* in local leaves has been reported (Vincent *et al.*, 2017; Nguyen *et al.*, 2018). Thus, we determined whether GLR3.3 and GLR3.6 are needed for the systemic resistance to piercing-sucking and chewing insects. Given that mechanical wounding in the Arabidopsis L8 led to systemic [Ca^2+^]_cyt_ response in leaves 5 and 13 (Kiep *et al.*, 2015), in this study we chose the eighth leaves (L8) as the local leaves and the 13th leaves (L13) as the systemic leaves for determination of systemic resistance.

**Fig. 2.**
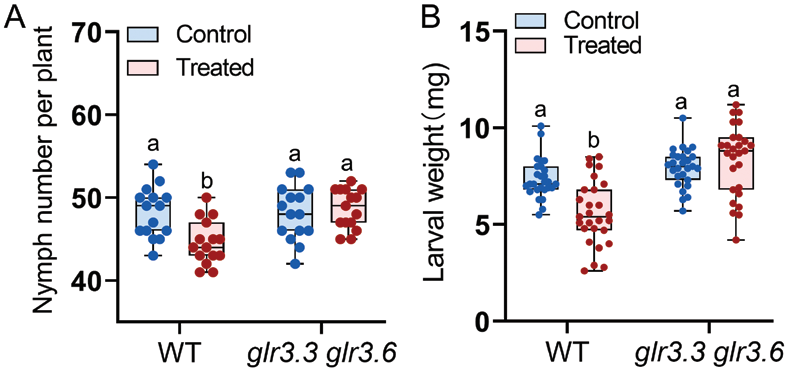
GLR3.3 and GLR3.6 are required for systemic resistance to GPA and CLW. (A) GPA feeding-induced resistance to GPA in systemic leaf. The eighth leaf (L8) was infested with 50 GPAs (treated) or mock-treated (no GPAs; control); after 3 d, the 13th systemic leaf (L13) was infested with one adult GPA, and the number of its offspring was recorded every day for 2 weeks (*n*=15). (B) Simulated CLW feeding-induced systemic resistance to CLW. L8 was treated with W+OS (treated) or untreated (control), and after 2 d, two freshly hatched CLW larvae were placed on the systemic L13; 1 d later, the biomasses of CLW larvae were recorded (*n*=27). In each box plot, the centre line locates the median, the box encompasses the upper and lower quartiles, each dot in the box plots indicates a specific sample, the error bars show the maximum and minimum of the distributions. Different letters indicate significant differences (two-way ANOVA followed by Tukey’s multiple comparisons test, *P*<0.05).

Fifty GPAs or no aphids (control) were infested on each of the L8 of WT and *glr3.3 glr3.6* plants; after 3 d, one adult GPA was infested on systemic L13 for another 14 d. On the WT plants, compared with the control group, the GPA nymph numbers on L13 decreased 6.4%, when L8 were treated with GPAs (Fig. 2A); however, on the *glr3.3 glr3.6* plants, the GPA nymph numbers on the L13 were not different between the control and GPA-infested group (Fig. 2A). When L8 of WT plants were treated with W+OS, the masses of CLWs on L13 decreased 21%, compared with the control group (Fig. 2B); on the *glr3.3 glr3.6* mutants, the larvae infested on the L13 had the same masses in both the control and W+OS-treated group (Fig. 2B). Previously it was shown that the *glr3.6* single mutants exhibited similar resistance levels to *Spodoptera littoralis* as the WT Arabidopsis, although the *glr3.3* and *glr3.3 glr3.6* mutants were susceptible (Nguyen *et al.*, 2018). We found that like the WT plants, the *glr3.3* and *glr3.6* mutants had normal systemic resistance to GPA and CLW (Supplementary Fig. S1), indicating that GLR3.3 and GLR3.6 play a redundant but essential role in systemic resistance to GPA and CLW insects.

### GLR3.3 and GLR3.6 are involved in regulation of systemic accumulation of JA and GSs

**Fig. 3.**
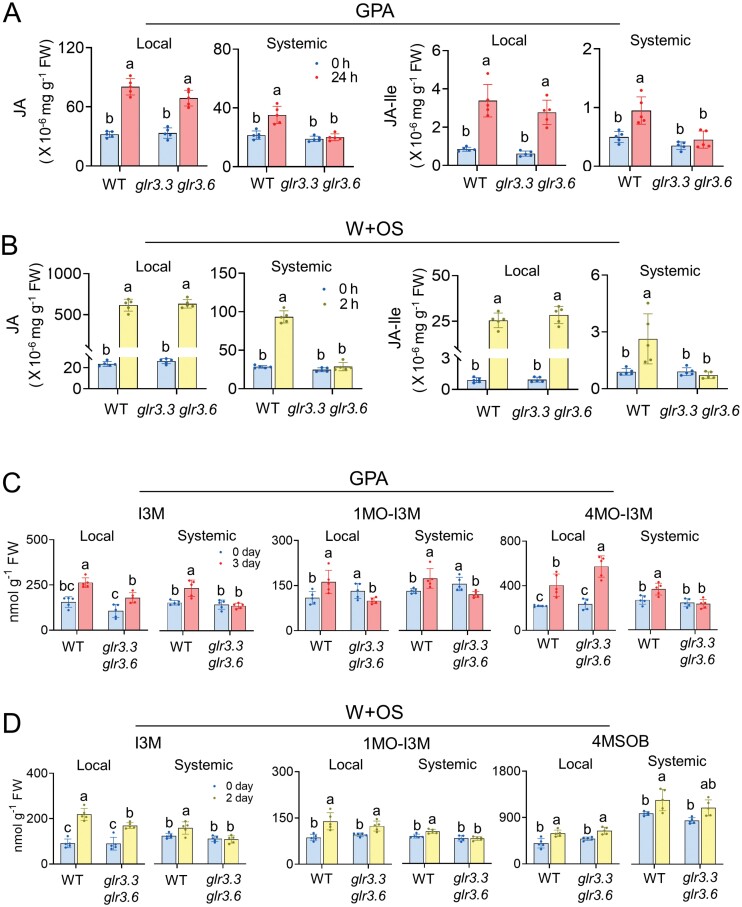
GPA feeding- and W+OS-induced JA, JA-Ile, and GSs in WT and *glr3.3 glr3.6* plants. Local leaves from WT and *glr3.3 glr3.6* plants were infested with 50 GPAs or treated with W+OS. Local and systemic leaves were harvested at the indicated times. (A, B) Concentrations of JA and JA-Ile in local and systemic leaves of WT and *glr3.3 glr3.6* plants in response to GPA feeding for 24 h (A) or2 h after W+OS treatment (B) (0 h = control; *n*=5). (C, D) The contents of GSs in local and systemic leaves of WT and *glr3.3 glr3.6* plants in response to GPA feeding for 3 d (C) or 2 d after W+OS treatment (D) (0 h = control; *n*=5). Values are means ±SD. Different letters indicate significant difference within the local or systemic dataset (two-way ANOVA followed by Tukey’s multiple comparisons test, *P*<0.05). The bars with blue colour represent the control group (0 h or 0 d), red ones represent the GPA treatment group (24 h or 3 d), and yellow ones represent the W+OS treatment group (2 h or 2 d). Local = L8, systemic = L13.

In order to understand whether phytohormones are involved in GLR3.3 and GLR3.6-mediated systemic defence against insects in Arabidopsis, we quantified the contents of phytohormones in the local and systemic leaves of WT and *glr3.3 glr3.6* plants. After 24 h of GPA infestation on L8, the JA and JA-Ile (jasmonoyl-isoleucine conjugate) contents increased in L8 of both WT (1.5- and 3-fold) and *glr3.3 glr3.6* (1.1- and 3.5-fold); in L13, JA and JA-Ile levels increased in the WT plants (64% and 90%), but not in the *glr3.3 glr3.6* plants (Fig. 3A). Compared with those in the control group, 2 h after W+OS treatment on L8, the JA and JA-Ile contents in L8 strongly increased in the WT (25.2- and 25.5-fold) and *glr3.3 glr3.6* plants (23- and 28.4-fold; Fig. 3B). Similar to GPA infestation, W+OS treatment did not induce systemic changes of JA and JA-Ile in *glr3.3 glr3.6*, whereas in L13 of WT plants, JA and JA-Ile increased 2.3- and 2.6-fold respectively (Fig. 3B). In addition, unlike JA and JA-Ile, ABA, SA, and IAA concentrations were similar in the WT and *glr3.3 glr3.6* plants, suggesting that GLR3.3 and GLR3.6 are not involved in regulating systemic ABA, SA, and IAA contents (Supplementary Fig. S2).

We hypothesized that loss of function of *GLR3.3* and *GLR3.6* might influence the systemic accumulation of GSs. Indeed, the contents of GSs showed a similar response as JA and JA-Ile. After GPA infestation on L8 for 3 d, compared with the control group, the contents of main indole-GSs I3M (Indol-3-ylmethyl-GS), 1MO-I3M (1-methoxyindol-3-ylmethyl-GS), and 4MO-I3M (4-methoxyindol-3-ylmethyl-GS) in L8 elevated 69%, 49%, and 85%, respectively in WT plants (Fig. 3C), and also elevated in the *glr3.3 glr3.6* mutants (68%, 27%, and 143%, respectively; Fig. 3C). In L13, the contents of I3M, 1MO-I3M and 4MO-I3M only increased in the WT plants (52%, 28%, and 36%, respectively) but not in the *glr3.3 glr3.6* mutants (Fig. 3C). Two days after W+OS treatment, in L8, the indole GSs (I3M and 1MO-I3M) and the aliphatic GS 4MSOB (4-methylsulfinylbutyl-GS) all increased in the WT (141%, 61%, and 48%, respectively) and *glr3.3 glr3.6* plants (89%, 29% and 32%, respectively; Fig. 3D); in L13, I3M and 1MO-I3M also accumulated in the WT plants (28% and 17%, respectively) but not in the *glr3.3 glr3.6* mutants (Fig. 3D). Notably, the contents of 4MSOB were not much affected by GLR3.3 and GLR3.6, since the 4MSOB content was elevated in L13 of both WT (27%) plants and *glr3.3 glr3.6* mutants (18%), after W+OS treatment on L8 (Fig. 3D). Consistent with the normal resistance to GPA and CLW in the *glr3.3* and *glr3.6* mutants, in response to GPA or simulated CLW feeding, the contents of JA and JA-Ile (Supplementary Fig. S3) and GSs (Supplementary Fig. S4), but not SA, IAA, and ABA (Supplementary Fig. S5), were elevated in the systemic leaves of *glr3.3* and *glr3.6* single mutants. ANOVA (analysis of variance) indicated that in the systemic leaves, GPA or W+OS always, while in many but not all cases, genotype affected the levels of JA, JA-Ile, and GSs (Supplementary Fig. S3, S4). These results indicate that GLR3.3 and GLR3.6 redundantly control GPA- and CLW feeding-induced accumulation of phytohormones JA and JA-Ile, and the defensive metabolites indole GSs in systemic leaves.

### GLR3.3 and GLR3.6 regulate insect feeding-induced transcriptome changes locally and systemically

To evaluate the function of GLR3.3 and GLR3.6 in regulation of plant defence-related gene expression on a genome-wide scale, we performed RNA-sequencing (RNA-seq) experiments. First, the differentially expressed genes (DEGs) were identified between the *glr3.3 glr3.6* and WT plants. Under the control conditions of the GPA feeding experiment, 2040 and 696 DEGs were found in local and systemic leaves, respectively, while 442 (local) and 624 (systemic) DEGs were identified after GPA feeding (Supplementary Fig. S6A, B; Supplementary Table S1). We found 207 and 209 DEGs in the local and systemic leaves, respectively, under the control conditions of W+OS experiment, and 720 (local) and 325 (systemic) DEGs were identified after W+OS treatment (Supplementary Fig. S7A, B; Supplementary Table S1). JA- and GS-related genes were found among these DEGs (Supplementary Figs S6, S7; Supplementary Table S1).

**Fig. 4.**
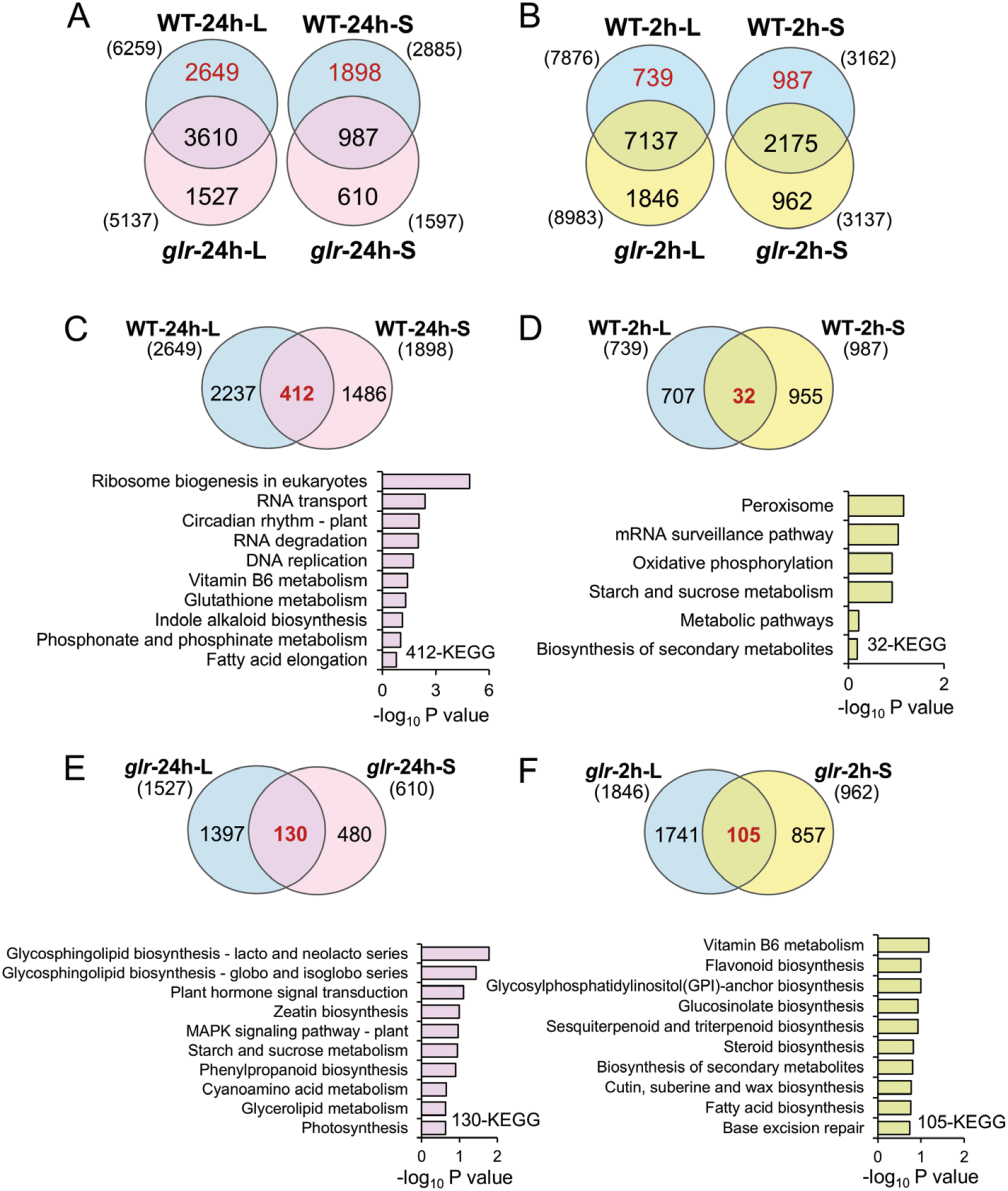
Changes in global transcript expression in local and systemic leaves of WT and *glr3.3 glr3.6* plants in response to GPA and W+OS treatment. WT and *glr3.3 glr3.6* (*glr*) plants were treated with GPA feeding for 24 h or W+OS for 2 h, and local (L) and systemic (S) leaves were harvested for RNA-seq analysis. (A, B) Venn diagrams indicating the numbers of common and specific DEGs identified in local and systemic leaves of *glr3.3 glr3.6* and WT plants in response to 24 h of GPA feeding (A) or 2 h after W+OS treatment (B). (C, D) Venn diagram analysis on the uniquely regulated DEGs between local and systemic leaves in WT plants after GPA feeding (C) and W+OS treatment (D). (E, F) Venn diagram analysis on uniquely regulated DEGs between local and systemic leaves in *glr3.3 glr3.6* plants after GPA feeding (E) and W+OS treatment (F). The bottom panels of (C) to (F) indicate the top 10 enriched pathways. The numbers in the brackets indicate the total numbers of DEGs. Complete data can be found in Supplementary Tables S2, S3, S6.

To investigate the function of GLR3.3 and GLR3.6 in regulating plant responses to herbivory, we next only focused on identifying the GPA- and W+OS-induced DEGs in WT and *glr3.3 glr3.6* plants. After GPA feeding for 24 h, 6259 and 5137 DEGs were identified in L8 of WT and *glr3.3 glr3.6* plants, respectively, among which 3610 genes were commonly regulated, while 2649 (42%) and 1527 (30%) unique DEGs were specific for WT and *glr3.3 glr3.6* plants, respectively (Fig. 4A, left; Supplementary Table S2); 2885 and 1597 DEGs were identified in L13 of WT and *glr3.3 glr3.6* plants, respectively, among which only 987 DEGs were common, and 1898 (66%) and 610 (38%) DEGs were unique for WT and *glr3.3 glr3.6,* respectively (Fig. 4A, right; Supplementary Table S2). Moreover, almost all the commonly regulated genes between WT and *glr3.3 glr3.6* in local (3610) and systemic (987) leaves showed the same directions of regulation (Supplementary Fig. S8A–D; Supplementary Table S2).

Two hours after W+OS treatment, 7876 and 8983 DEGs were identified in L8 of WT and *glr3.3 glr3.6* plants, respectively, and most DEGs (7137) were co-regulated (Fig. 4B, left; Supplementary Table S3). We found similar changes in DEG numbers in L13 of WT (3162) and *glr3.3 glr3.6* (3137) plants, and 2175 DEGs were commonly regulated, while 987 (31%) and 962 (31%) DEGs were unique for the WT and *glr3.3 glr3.6* plants, respectively (Fig. 4B, right; Supplementary Table S3). Again, among the commonly regulated genes between WT and *glr3.3 glr3.6* plants, only six (local) and three (systemic) genes were found to be regulated in opposite directions in the local and systemic leaves, respectively (Supplementary Fig. S8E, F; Supplementary Table S3). Thus, greater portions of DEGs were specifically regulated by GLR3.3 and GLR3.6 in the systemic leaves than in the local leaves in Arabidopsis in response to either GPA or simulated CLW infestation, suggesting that GLR3.3 and GLR3.6 play a more important role in regulating systemic responses than local responses.

Next, we performed Kyoto Encyclopedia of Genes and Genomes (KEGG) enrichment analysis on these uniquely regulated DEGs in both WT and *glr3.3 glr3.6* plants (Fig. 4A, B), since the DEGs specific for the WT plants are those requiring GLR3.3 and GLR3.6 for their transcriptional regulation, while the regulation of DEGs specific for the *glr3.3 glr3.6* mutants were normally repressed by GLR3.3 and GLR3.6. The pathways related to the metabolism of amino acids and carbohydrates were enriched for these DEGs, regardless of GPA or W+OS treatment (Supplementary Figs S9–S12; Supplementary Tables S4, S5), suggesting the potentially important role of GLR3.3 and GLR3.6 in controlling herbivory-induced reconfiguration of metabolites and the underlying transcripts/pathways, including amino acids and carbohydrates.

Furthermore, the 2649 and 1898 DEGs identified local and systemic leaves, respectively, in the WT plants, in response to GPA feeding, whose expression was dependent on GLR3.3 and GLR3.6, were used for Venn diagram analysis: we identified 412 genes (Fig. 4C). KEGG analysis indicated that the enriched pathways from these 412 genes included ‘ribosome biogenesis in eukaryotes’, ‘RNA transport’, ‘vitamin B6 metabolism’, and ‘glutathione metabolism’ (Fig. 4C; Supplementary Table S6). In contrast, only 32 GLR3.3 and GLR3.6-regulated genes were commonly identified from the 739 and 987 DEGs in the local and systemic leaves, respectively, of WT plants in response to W+OS (Fig. 4D), and ‘peroxisome’, ‘mRNA surveillance pathway’, ‘oxidative phosphorylation’, ‘starch and sucrose metabolism’, ‘metabolic pathways’, and ‘biosynthesis of secondary metabolites’ were enriched (Fig. 4D; Supplementary Table S6). Additionally, to identify the genes whose regulation is repressed by GLR3.3 and GLR3.6, 1527 (local) and 610 (systemic) DEGs and the 1846 (local) and 962 (systemic) DEGs specific for the *glr3.3 glr3.6* mutants in response to GPA and W+OS feeding (Fig. 4A, B), respectively, were also used for Venn diagram analysis: 130 (GPA) and 105 (W+OS) DEGs were identified (Fig. 4E, F). ‘Glycosphingolipid biosynthesis’, ‘plant hormone signal transduction’, and ‘MAPK signalling pathway’ were among the pathways enriched from the 130 genes; ‘flavonoid biosynthesis’, ‘GS biosynthesis’, and ‘biosynthesis of secondary metabolites’ were among the pathways enriched from the 105 genes (Fig. 4E, F; Supplementary Table S6).

Next, we sought to identify genes that were regulated in both local and systemic leaves and by both GPA and W+OS treatment, and these genes could be considered the core responsive genes to both GPA and CLW. Venn diagram analysis on the GLR3.3- and GLR3.6-dependent DEGs regulated by GPA and W+OS in the local (2649 and 739, respectively; Fig. 4A, B) and systemic leaves (1898 and 987, respectively; Fig. 4A, B) resulted in 125 (local) and 146 (systemic) common genes (Supplementary Fig. S13A, B). The enriched pathways from the former 125 genes included ‘GS biosynthesis’, ‘alanine, aspartate, and glutamate metabolism’, and those enriched from the latter 146 genes included ‘cyanoamino acid metabolism’, ‘starch and sucrose metabolism’, and ‘phenylpropanoid biosynthesis’ (Supplementary Fig. S13C, D; Supplementary Table S7). Similarly, Venn diagram analysis on the GLR3.3- and GLR3.6-repressed DEGs, in response to GPA and W+OS in the local (1527 and 1846, respectively; Fig. 4A, B) and systemic leaves (610 and 962, respectively; Fig. 4A, B) resulted in 170 (local) and 45 (systemic) common genes (Supplementary Fig. S14A, B; Supplementary Table S7). The enriched pathways from the former 170 genes included several pathways related to amino acid metabolism, and those enriched from the latter 45 genes included ‘plant hormone signal transduction’, ‘sphingolipid metabolism’, and ‘MAPK signaling’ (Supplementary Fig. S14C, D; Supplementary Table S7). Furthermore, most of these genes showed the same directions of regulation in response to GPA and W+OS treatment (Supplementary Figs S13E, F, S14E, F; Supplementary Table S7).

Given the important roles of JA and GSs in plant defence against insects, we specifically inspected the JA- and GS-related genes in the transcriptome data. In the local leaves after GPA feeding but not W+OS treatment, while in the systemic leaves after both GPA and W+OS treatment, most of the JA- and GS-related genes in the *glr3.3 glr3.6* double mutant exhibited opposite directions of regulation from the same genes in the WT plants (Supplementary Figs S15, S16; Supplementary Table S8, S9). Thus, GPA and W+OS induced the expression of JA- and GS-related genes in both local and systemic leaves, and their expression is at least partially dependent on GLR3.3 and GLR3.6, especially in the systemic leaves.

### The *glr3.3 glr3.6* mutants show substantially altered metabolomes

Given the important role of GLR3.3 and GLR3.6 in regulating the transcriptome, including the pathways related to the metabolism of amino acids and carbohydrates, we speculated that GLR3.3 and GLR3.6 might also control the accumulation of certain metabolites. Under the control conditions, between the WT and *glr3.3 glr3.6* plants, there were 32 (local) and 51 (systemic) differentially produced metabolites (DPMs), respectively; while after GPA feeding, 18 (local) and 26 (systemic) DPMs were identified (Supplementary Fig. S17A, B; Supplementary Table S10). When not being treated with W+OS, between the WT and *glr3.3 glr3.6* plants, 58 (local) and 48 (systemic) DPMs were identified, respectively; after W+OS treatment, 94 (local) and 77 (systemic) DPMs were respectively found (Supplementary Fig. S18A, B; Supplementary Table S10). These DPMs included organic acids and carbohydrates (Supplementary Figs S17C, D, S18C, D; Supplementary Table S10).

**Fig. 5.**
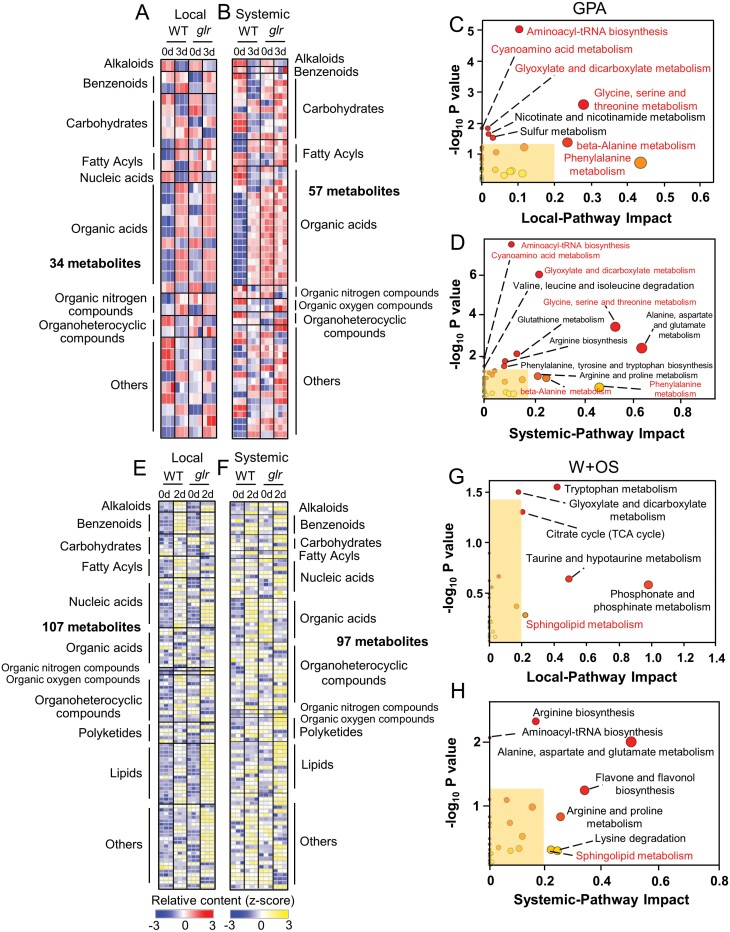
Differentially regulated metabolites in the local and systemic leaves of WT and *glr3.3 glr3.6* plants in response to GPA infestation and W+OS treatment. (A, B) Heatmaps indicating the relative contents of DRMs in (A) local and (B) systemic leaves of WT and *glr3.3 glr3.6* plants after 3 d of GPA feeding. (C, D) Metabolic pathway analysis of the 34 and 57 DRMs shown in (A) and (B). The pathways in red indicate the common ones in both local and systemic leaves. (E, F) Heatmaps indicating the relative contents of differentially regulated metabolites in local (E) andsystemic leaves (F) of WT and *glr3.3 glr3.6* plants 2 d after W+OS treatment. (G, H) Metabolic pathway analysis of the 107 and 97 DRMs shown in (E) and (F). The pathways in red indicate the common ones in both local and systemic leaves. (A), (B), (E), (F): Relative contents of metabolites were first log_2_ transformed and then normalized using the Z-score algorithm. Each block represents a biological replicate (*n*=3). (C), (D), (G), (H): *P*-values reflect the over-representation of each category, while the pathway impact weighs the importance of the affected metabolites within the pathway; each circle marks a pathway, and larger sizes and darker colours suggest higher pathway impact and greater pathway enrichment (lower *P*-values). Yellow boxes highlight pathway whose impacts <0.2 and statistically not significant (*P*>0.05, hypergeometric test). Complete data can be found in Supplementary Tables S13, S14.

To gain insight into the regulatory function of GLR3.3 and GLR3.6 in modulating changes of metabolites, we next focused on how WT and *glr3.3 glr3.6* plants respond to GPA feeding and W+OS treatment. In the GPA feeding-treated WT and *glr3.3 glr3.6* plants, 58 and 56 DPMs, respectively, were found in L8 (Supplementary Fig. S19A), and 58 and 34 DPMs, respectively, were identified in L13 (Supplementary Fig. S19B; Supplementary Table S11). After W+OS treatment, in L8 of WT and *glr3.3 glr3.6* plants, we found 193 and 210 DPMs, respectively, while in L13, there were 60 and 84 DPMs, respectively (Supplementary Fig. S20; Supplementary Table S12). Then we specifically focused on the metabolites in the local and systemic leaves which exhibited differential regulation between WT and *glr3.3 glr3.6* plants [differentially regulated metabolites (DRMs)]. After GPA feeding for 3 d, compared with the WT plants, 34 and 57 DRMs were found in the local and systemic leaves, respectively, of *glr3.3 glr3.6* mutants, and these metabolites mainly included carbohydrates and organic acids (Supplementary Fig. 5A, B; Supplementary Table S13). Hypergeometric tests revealed that eight and 12 metabolic pathways were controlled by GLR3.3 and GLR3.6, respectively, in the local and systemic leaves in response to GPA feeding (Fig. 5C, D; Table 13), among which six pathways were common. Most of these common pathways are related to amino acid metabolism, suggesting that the metabolism of amino acids is controlled by GLR3.3 and GLR3.6 in both local and systemic leaves (Fig. 5C, D; Table 13).

Two days after W+OS treatment, compared with the WT plants, we found 107 and 97 DRMs in the local and systemic leaves, respectively, of *glr3.3 glr3.6* mutants (Fig. 5E, F; Supplementary Table S14). Most of these metabolites are carbohydrates, nucleic acids, organic acids, organoheterocyclic compounds, polyketides, and lipids (Fig. 5E, F; Supplementary Table S14). In addition, only ‘sphingolipid metabolism’ pathway was regulated by GLR3.3 and GLR3.6 in both local and systemic leaves (Fig. 5G, H). Five pathways were enriched from the DRMs between the local leaves of WT and *glr3.3 glr3.6* plants, including two carbohydrate metabolism pathways (Fig. 5G; Supplementary Table S14); six pathways were enriched only in the systemic leaves, which included four pathways of amino acid metabolism (Fig. 5H; Supplementary Table S14).

**Fig. 6.**
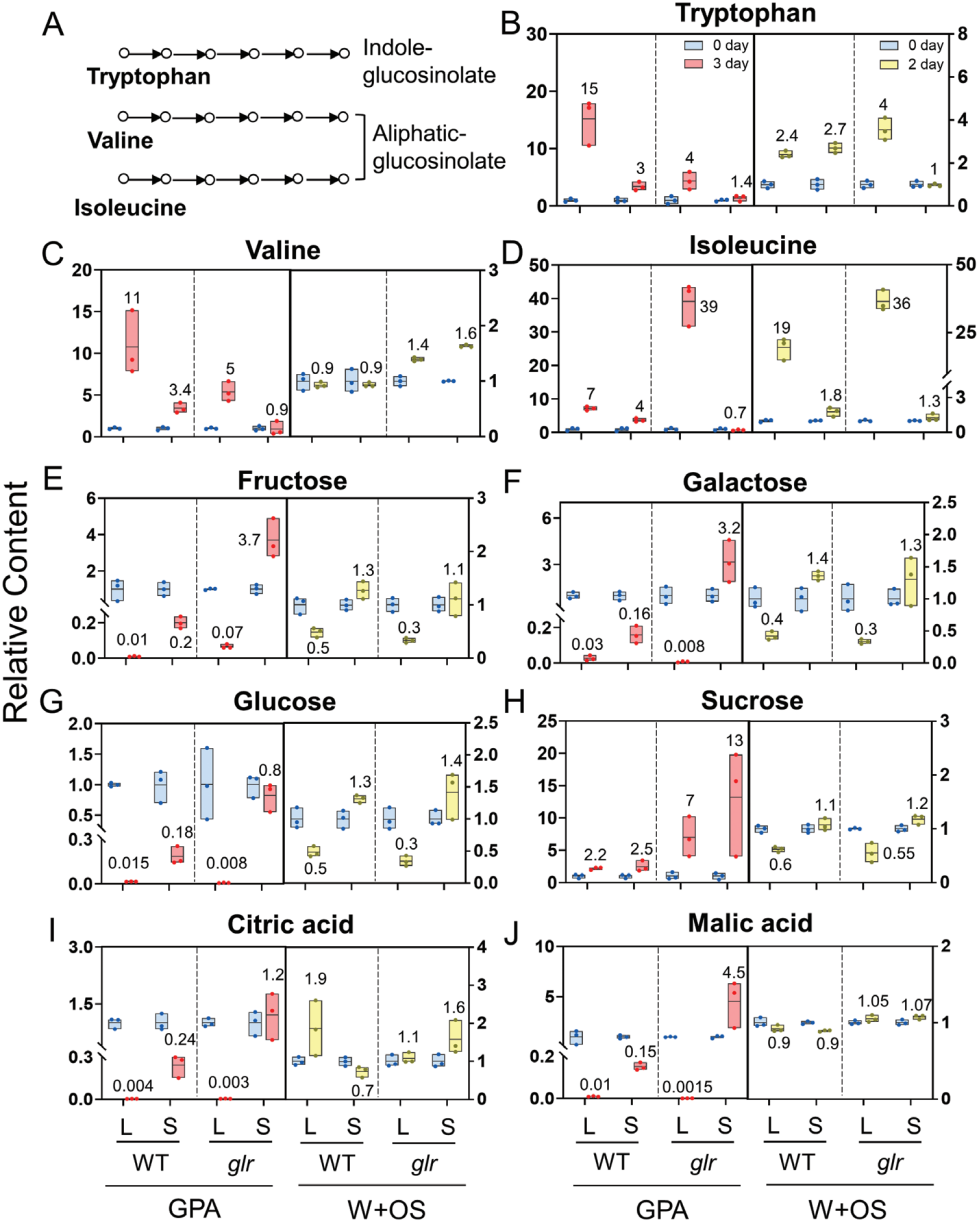
The relative content of specific amino acids, sugars, and organic acids in WT and *glr3.3 glr3.6* plants in response to GPA infestation and W+OS treatment. (A) Schematic diagram illustrating the involvement of tryptophan, valine, and isoleucine in the GS biosynthesis pathway. WT and *glr3.3 glr3.6* (*glr*) plants were treated with GPA feeding (3 d) or W+OS (2 d). The relative content of amino acids (B) tryptophan, (C) valine, and (D) isoleucine, sugars (E) fructose, (F) galactose, (G) glucose, and (H) sucrose, and organic acids (I) citric acid and (J) malic acid in local (L) and systemic (S) leaves of WT and *glr3.3 glr3.6* plants. Blue bars are controls, red bars indicate samples treated with GPA feeding for 3 d, and the yellow bars indicate samples treated with W+OS treatment for 2 d. Numbers next to the red or yellow bars are the fold change values (relative content in treatment group/average relative content in control group). Floating bar represent minimum and maximum range of variation; the centre line indicates the mean; each dot indicates a specific sample (*n*=3). Complete data can be found in Supplementary Table S15.

GSs in Arabidopsis are mostly derived from tryptophan, valine, and isoleucine (Wittstock and Halkier, 2002; Fig. 6A). Thus, the relative content of tryptophan, valine, and isoleucine were specifically inspected by metabolomic HPLC-MS/MS analysis. In the WT plants, GPA feeding elicited a strong increase in the indole-GS precursor tryptophan not only in the local leaves (14-fold) but also in the systemic leaves (2-fold), while in the *glr3.3 glr3.6* mutants, its contents were elevated in the local leaves (3-fold) but only slightly changed in the systemic leaves (40% increase; Fig. 6B, left; Supplementary Table S15). This is in line with our results showing GPA feeding-induced indole-GS content in the systemic leaves of WT plants, but not in the *glr3.3 glr3.6* plants (Fig. 3C). The contents of aliphatic-GS precursor valine and isoleucine were elevated in both local (10- and 6-fold, respectively) and systemic (2.4- and 3-fold, respectively) leaves of WT plants, and their contents were also up-regulated (4- and 38-fold, respectively) in the local leaves of *glr3.3 glr3.6* mutants, but not in the systemic leaves (Fig. 6C, D, left; Supplementary Table S15). Thus, GLR3.3 and GLR3.6 play important roles in mediating the local and systemic accumulation of tryptophan, valine, and isoleucine in Arabidopsis defence against GPA. Similarly, after W+OS treatment, in the WT plants, the content of tryptophan and isoleucine were induced in both local (1.4- and 18-fold, respectively) and systemic leaves (1.7- and 0.8-fold, respectively; Fig. 6B, D, right; Supplementary Table S15). In the *glr3.3 glr3.6* mutants, tryptophan and isoleucine increased (3- and 35-fold, respectively) in the local leaves, while their contents were not altered in the systemic leaves (Fig. 6B, D, right; Supplementary Table S15). Valine content was not obviously affected by W+OS in either local or systemic leaves of WT or *glr3.3 glr3.6* plants (Fig. 6C, right; Supplementary Table S15).

In addition, we observed strong alteration in the carbohydrate content when plants were subjected to GPA feeding or W+OS treatment. After GPA feeding, fructose, galactose, and glucose all greatly decreased in the local leaves of WT (99%, 97%, and 98.5%, respectively) and *glr3.3 glr3.6* plants (93%, 99.2%, and 99.2%, respectively; Fig. 6E-G, left; Supplementary Table S15); in the systemic leaves of WT plants, the content of fructose, galactose, and glucose also greatly dropped (80%, 84%, and 82%, respectively), but in the *glr3.3 glr3.6* mutants, fructose and galactose increased 2.7- and 2.2-fold, respectively, and glucose decreased 20% (Fig. 6E, G, left; Supplementary Table S15). Moreover, sucrose content was up-regulated in both local and systemic leaves of WT plants (1.2- and 1.5-fold, respectively), but were even more highly up-regulated in the *glr3.3 glr3.6* plants (6- and 12-fold, respectively; Fig. 6H, left; Supplementary Table S15). In response to W+OS, the contents of fructose, galactose, glucose, and sucrose were similarly decreased in the local leaves of WT and *glr3.3 glr3.6* plants, but their contents were unchanged in the systemic leaves of both plants (Fig. 6E, H, right; Supplementary Table S15). Citric acid and malic acid are the intermediates of the TCA cycle, and they are central to numerous interrelated metabolic networks that produce a myriad of secondary metabolites, amino acids, and phytohormones (Araujo *et al.*, 2014; Tahjib-Ul-Arif *et al.*, 2021). After GPA feeding, the contents of citric acid and malic acid also markedly declined in the local leaves of both WT (99.6% and 99%, respectively) and *glr3.3 glr3.6* plants (99.7% and 99.85%, respectively) (Fig. 6I, J, left; Supplementary Table S15), and similar to sugars, their contents decreased in the systemic leaves of WT plants (76% and 85%, respectively) but not *glr3.3 glr3.6* mutants (Fig. 6I, J, left). In contrast, after W+OS treatment, these two organic acids exhibited no obvious changes in both WT and *glr3.3 glr3.6* plants regardless of local or systemic leaves (Fig. 6I, J, right; Supplementary Table S15).

Thus, our data show that amino acids, carbohydrates, and organic acids are highly regulated in both local and systemic leaves after insect feeding in an insect species-specific manner, and the reconfiguration of the metabolism of these compounds in local and systemic leaves, especially in systemic leaves, is regulated at least partly by GLR3.3 and GLR3.6.

## Discussion

Systemic responses, controlled by a complex network of cell-to-cell communication, are important for plant adaption to biotic and abiotic stresses. The ROS wave, calcium (Ca^2+^) wave, and electrical signals are three known players of this complex network of cell-to-cell communication (Choi *et al.*, 2017). Wounding- or insect herbivory-induced systemic defence was discovered 50 years ago (Green and Ryan, 1972); however, how local and systemic tissues communicate upon herbivory, including herbivory from different types of insects, remain unclear. Elegant studies have revealed that GLR3.3 and GLR3.6, two glutamate receptor-like channels, mediate wounding-induced long-distance electrical and Ca^2+^ signals between local and systemic leaves (Mousavi *et al.*, 2013; Nguyen *et al.*, 2018; Toyota *et al.*, 2018). Here, we demonstrated that GLR3.3 and GLR3.6 are important for GPA and simulated CLW feeding-induced systemic Ca^2+^ signalling, and confer systemic resistance to GPA and CLW likely by regulating the contents of JA and GSs in systemic leaves.

GLR3.3 and GLR3.6 are clearly central players in wounding- and herbivory-induced systemic Ca^2+^ signalling (Toyota *et al.*, 2018). In this study, we found that GLR3.3 and GLR3.6 also support long-distance transmission of GPA-induced [Ca^2+^]_cyt_ increases (Fig. 1). A previous study demonstrated that the larvae of *Spodoptera littoralis* gained weight faster on the *glr3.3 glr3.6* mutants than on WT Arabidopsis plants (Nguyen *et al.*, 2018), but the fecundity of GPA on the *glr3.3 glr3.6* mutants was not different from that of GPA on the WT plants (Vincent *et al.*, 2017). Here we focused on the systemic resistance and found that only the *glr3.3 glr3.6* double mutants, but not the *glr3.3* and *glr3.6* single mutants, had impaired systemic resistance to GPAs and CLWs (Fig. 2; Supplementary Fig. S1). We found that either GPA or simulated CLW feeding (W+OS) induced increased levels of JA, JA-Ile, and GSs in systemic leaves of WT plants, and the *glr3.3* and *glr3.6* single mutants, but not the *glr3.3 glr3.6* mutants (Fig. 3; Supplementary Figs S3, S4), indicating that GLR3.3 and GLR3.6 redundantly play an essential role in regulating systemic accumulation of JA and GSs.

We proposed that GLR3.3 and GLR3.6 may affect the levels of JA and GSs at least partly through regulating the expression of JA- and GS-related biosynthetic genes during systemic response to herbivory attack. Using transcriptome analysis, we show that GLR3.3 and GLR3.6 regulate the GPA feeding-induced JA- and GS-related genes in both local and systemic leaves, and also play a more important role in regulating W+OS-induced JA- and GS-related genes in systemic leaves than in local leaves (Supplementary Figs S15, S16). Previous studies have demonstrated that wounding induced similar expression levels of JA biosynthesis gene *OPR3* (*OPDA reductase 3*), and jasmonate-ZIM repressors *JAZ5*, *JAZ7*, and *JAZ10* in the local leaves of WT and *glr3.3 glr3.6*, while the expression of these genes was severely suppressed in the systemic leaves of *glr3.3 glr3.6* mutants (Mousavi *et al.*, 2013; Fichman and Mittler, 2021). Furthermore, GLR3.3- and GLR3.6-regulated Ca^2+^ signalling may directly affect JA biosynthesis. For example, the JA biosynthesis enzyme LOX6 (lipoxygenase 6) was shown to be the major contributor to wounding-induced JA in Arabidopsis systemic leaves (Chauvin *et al.*, 2013). Moreover, most LOXs have putative Ca^2+^ domains, and Ca^2+^ binding can stimulate their enzyme activity (Oldham *et al.*, 2005; Farmer *et al.*, 2014). Thus, the Ca^2+^ fluxes which are controlled directly or indirectly by GLR3.3 and GLR3.6 may activate plant LOXs to stimulate *de novo* JA synthesis (Farmer *et al.*, 2014).

Nguyen *et al.* (2018) revealed that GLR3.3 and GLR3.6 also function in wound-activated electrical signal propagation between leaves. More recently, Fichman and Mittler (2021) found that in response to wounding, systemic ROS level is also controlled by GLR3.3 and GLR3.6. It is likely that upon GPA feeding or W+OS treatment, GLR3.3 and GLR3.6 modulate the transmission of Ca^2+^, ROS, and electrical signals from local to the distal systemic leaves, thus activating JA biosynthesis transcriptionally and post-transcriptionally. Genetic analysis indicated that JA signalling plays an important role in regulating accumulation of GSs (Schweizer *et al.*, 2013). It is still unclear whether accumulation of JA is essential for the increase of GSs in systemic leaves. It would be interesting to study what the primary signal is in mediating JA accumulation in systemic leaves during herbivory, and how GLR3.3- and GLR3.6-mediated Ca^2+^, ROS, and electrical signals control JA biosynthesis. Furthermore, a P-type H^+^-ATPase AHA1 functions in wounding-activated long-distance electrical signal transmission and acts as a negative regulator of JA synthesis (Kumari *et al.*, 2019). A recent study also revealed that GLR3.3, GLR3.6, and AHA1 regulate wounding-induced systemic signals transferred from root to shoot in Arabidopsis, and it was proposed that after wounding, AHA1 activity results in apoplastic alkalinization; together with wounding-induced glutamate released into the apoplastic space, GLRs are activated, causing influx of Ca^2+^ and other cations through GLRs, thereby depolarizing the membranes (Shao *et al.*, 2020). Whether AHA1 is involved in herbivory-induced leaf-leaf systemic signalling merits further study.

Our transcriptome analysis provides a global view on the function of GLR3.3 and GLR3.6 in transcriptionally regulating plant defence against GPA and CLW: about 42% (2649/6259) and 9% (739/7876) of DEGs in local leaves were dependent on GLR3.3 and GLR3.6 after GPA and W+OS treatment, respectively, and 66% (1898/2885) and 31% (987/3162) of DEGs in systemic leaves were dependent on GLR3.3 and GLR3.6 after GPA and W+OS treatment, respectively (Fig. 4A, B). Therefore, GLR3.3 and GLR3.6 play a more important role in modulating transcriptomic responses to insect feeding in systemic leaves than in local leaves. Notably, KEGG analysis indicated that many of the GLR3.3- and GLR3.6-dependent genes are involved in various pathways, among which we found hormone signal transduction and the regulation of metabolism of secondary metabolites, including glucosinolates (consistent with our chemical analysis), and primary metabolites, including amino acids and carbohydrates (Fig. 4C-F; Supplementary Figs S9-S14). Moreover, these data strongly suggest that besides GLR3.3 and GLR3.6, there are other proteins that function in the regulation of plant systemic transcriptomic response to herbivore attack. Grafting is generally considered to be the best tool to study local-systemic signalling (Tsutsui and Notaguchi, 2017), and grafting experiments and genetic analyses are needed to further dissect the roles of ROS, Ca^2+^, JA, and electrical signalling, among others, in systemic signalling, and whether there are genetic interactions among them.

Through metabolome analysis, we show that GLR3.3 and GLR3.6 play an important role in regulating both local and systemic metabolites: (i) about 10% (6/58) and 52% (30/58) of GPA-induced DPMs in local and systemic leaves, respectively, and about 18% (34/193) and 57% (34/60) of W+OS-induced DPMs in local and systemic leaves, respectively, were completely dependent on GLR3.3 and GLR3.6 (Supplementary Figs S19C, S20C); (ii) after GPA feeding, 34 (local) and 57 (systemic) metabolites, and after W+OS treatment 107 (local) and 97 (systemic) metabolites showed obvious differential regulation patterns between the *glr3.3 glr3.6* mutants and WT plants (Fig. 5). Specifically, in systemic leaves, GLR3.3 and GLR3.6 modulate herbivory-induced amino acids tryptophan, valine, and isoleucine, which are the precursors of GSs (Wittstock and Halkier, 2002; Fig. 6A-D), and this is consistent with the results of GS content (Fig. 3C, D). Thus, in addition to transcriptionally regulating GS biosynthetic genes, GLR3.3 and GLR3.6 are also required for the normal accumulation of the precursor amino acids of GSs, and these may both contribute to the herbivory-induced accumulation of GSs in the systemic leaves.

Reduced photosynthetic activity was detected in greenbug aphid (*Schizaphis graminum*)-attacked sorghum and Colorado potato beetle (*Leptinotarsa decemlineata*)-infested potato plants (Zhu-Salzman *et al.*, 2004; Lawrence *et al.*, 2008). Yang *et al.* (2019) found that the contents of sucrose, fructose, and glucose decreased in both local and systemic leaves after leaf herbivory by the Lepidopteran insect *Ectropis oblique* on tea plants (*Camellia sinensis*). In this study, we show that after GPA feeding, fructose, galactose, and glucose were highly down-regulated in both local and systemic leaves of WT plants; however, in *glr3.3 glr3.6* mutants, these sugars were similarly down-regulated in local leaves, while in the systemic leaves fructose and galactose were up-regulated, and glucose was only slightly down-regulated (Fig. 6E-G); sucrose was moderately increased in the GPA-infested local and systemic leaves of WT plants; however, knocking out *GLR3.3* and *GLR3.6* led to even highly elevated sucrose content in all leaves (Fig. 6H). In contrast, compared with GPA herbivory, simulated CLW feeding did not have such strong effects on all the sugars detected. Thus, these data indicate the important role of GLR3.3 and GLR3.6 in regulating sugars in systemic leaves during Arabidopsis defence against GPA. 

Aphids insert their stylets to plant phloem tissue to feed on phloem sap, which contains high concentrations of sugars. Strong down-regulation of sugar content could be a strategy of defence against GPA, even though this hypothesis needs to be confirmed. Chewing insects consume the whole leaf lamina, and they generally do not directly contact the sugars in the phloem, and this might account for the fact that simulated CLW feeding did not have a strong impact on the sugars. In response to simulated *Manduca sexta* feeding, the wild tobacco *Nicotiana attenuata* increases the allocation of sugars to roots (Schwachtje *et al.*, 2006). The ecological significance of such extensive sugar down-regulation, and the target sites of the reallocated sugars remain to be studied.

Taken together, at the transcriptome and metabolome level, this study provides a comprehensive and global view on the role of GLR3.3 and GLR3.6 in plant systemic defence against both piercing-sucking and chewing insects. Our data also suggest that not only certain unknown regulators involved in systemic signalling also exist, but local-systemic signalling is very complex, involving various factors. Much research on Ca^2+^, ROS, electrical signalling, pH, GLRs, as well as other factors is needed for understanding the communications between local and systemic leaves.

## Supplementary data

The following supplementary data are available at *JXB* online. 

Video S1. W+OS-elicited [Ca^2+^]_cyt_ elevation in systemic leaves of WT plants.

Video S2. W+OS-elicited [Ca^2+^]_cyt_ elevation in systemic leaves of *glr3.3 glr3.6* mutants.

Video S3. Aphid herbivory-elicited [Ca^2+^]_cyt_ elevation in systemic leaves of WT plants.

Video S4. Aphid herbivory-elicited [Ca^2+^]_cyt_ elevation in systemic leaves of *glr3.3 glr3.6* mutants.

Fig. S1. The systemic resistance to GPA and CLW in WT plants, *glr3.3* and *glr3.6* single mutants, and *glr3.3 glr3.6* double mutants.

Fig. S2. Quantification of SA, ABA, and IAA in WT and *glr3.3 glr3.6* plants in response to GPA and W+OS treatment.

Fig. S3. Quantification of JA and JA-Ile in WT plants, *glr3.3* and *glr3.6* single mutants, and *glr3.3 glr3.6* double mutants in response to GPA and W+OS treatment.

Fig. S4. Quantification of GSs in WT, *glr3.3* and *glr3.6* single mutants, and *glr3.3 glr3.6* double mutants in response to GPA and W+OS treatment.

Fig. S5. Quantification of SA, ABA, and IAA in WT plants, *glr3.3* and *glr3.6* single mutants, and *glr3.3 glr3.6* double mutants in response to GPA and W+OS treatment.

Fig. S6. DEGs in local and systemic leaves of *glr3.3 glr3.6* mutants compared with WT under control conditions, and after GPA feeding for 24 h.

Fig. S7. DEGs in local and systemic leaves of *glr3.3 glr3.6* mutants compared with WT plants under control conditions, and 2 h after W+OS treatment.

Fig. S8. Common DEGs identified in WT and *glr3.3 glr3.6* plants which had opposite directions of regulation.

Fig. S9. KEGG enrichment analysis on the DEGs in local leaves of WT and *glr3.3 glr3.6* plants in response to GPA infestation.

Fig. S10. KEGG enrichment analysis on DEGs in systemic leaves of WT and *glr3.3 glr3.6* plants in response to GPA infestation.

Fig. S11. KEGG enrichment analysis on DEGs in local leaves of WT and *glr3.3 glr3.6* plants in response to W+OS treatment.

Fig. S12. KEGG enrichment analysis on DEGs in systemic leaves of WT and *glr3.3 glr3.6* plants in response to W+OS treatment.

Fig. S13. The uniquely regulated DEGs in local and systemic leaves of WT plants in response to GPA feeding and W+OS treatment.

Fig. S14. Uniquely regulated DEGs in local and systemic leaves of *glr3.3 glr3.6* mutants in response to GPA and W+OS treatment.

Fig. S15. Relative transcript levels of genes involved in JA biosynthesis, metabolism, and perception.

Fig. S16. Relative transcripts level of genes involved in GS biosynthesis, activation, regulation, and transport.

Fig. S17. DPMs in local and systemic leaves of *glr3.3 glr3.6* mutants compared with WT plants under control conditions, and after GPA feeding for 3 d.

Fig. S18. DPMs in local and systemic leaves of *glr3.3 glr3.6* mutants compared with WT plants under control conditions, and after W+OS treatment for 2 d.

Fig. S19. Relative content of differentially produced metabolites in the local and systemic leaves of WT and *glr3.3 glr3.6* plants following GPA infestation.

Fig. S20. Relative content of differentially produced metabolites in the local and systemic leaves of WT and *glr3.3 glr3.6* plants following W+OS treatment.

Table S1. DEGs in local and systemic leaves of *glr3.3 glr3.6* plants compared with WT before and after GPA or W+OS treatment.

Table S2. DEGs of WT and *glr* after GPA feeding.

Table S3. DEGs of WT and *glr* after W+OS treatment.

Table S4. KEGG analysis of uniquely regulated genes in WT and *glr* after GPA feeding.

Table S5. KEGG analysis of uniquely regulated genes in WT and *glr* after W+OS treatment.

Table S6. Analysis of co-regulated genes between local and systemic leaves of WT or *glr3.3 glr3.6* under two treatments.

Table S7. Transcript analysis of local and systemic leaves of WT and *glr3.3 glr3.6* plants between GPA and W+OS treatment.

Table S8. VST of transcripts in WT and *glr* after GPA feeding.

Table S9. VST of transcripts in WT and *glr* after W+OS treatment.

Table S10. DPMs in local and systemic leaves of *glr3.3 glr3.6* compared with WT plants before and after GPA or W+OS treatment.

Table S11. DPMs of WT and *glr* after GPA feeding.

Table S12. DPMs of WT and *glr* after W+OS treatment.

Table S13. DRMs of WT and *glr* after GPA feeding.

Table S14. DRMs of WT and *glr* after W+OS treatment.

Table S15. Fold change of representative metabolites.

erac399_suppl_Supplementary_Figures_S1-S20

erac399_suppl_Supplementary_Tables_S1-S3

erac399_suppl_Supplementary_Tables_S4-S5

erac399_suppl_Supplementary_Tables_S6-S7

erac399_suppl_Supplementary_Tables_S8-S9

erac399_suppl_Supplementary_Tables_S10-S12

erac399_suppl_Supplementary_Tables_S13-S15

erac399_suppl_Supplementary_Videos_S1-S4

## Data Availability

All the transcriptome data can be accessed at the National Genomics Data Center (https://bigd.big.ac.cn/) under BioProject ID PRJCA003953 and PRJCA005260.

## Abbreviations

CLW: cotton leafworm (*Spodoptera litura*)
GPA: green peach aphid (*Myzus persicae*)
GSs: glucosinolates
JA-Ile: (+)-7-*iso*-jasmonoyl-L-isoleucine
JA: jasmonic acid
