# Supplementary material for: The glutamate receptor-like 3.3 and 3.6 mediate systemic resistance to insect herbivores in Arabidopsis
Source: J Exp Bot. 2022 Oct 10;73(22):7611–27. doi: 10.1093/jxb/erac399 (PMC9730813; doi:10.1093/jxb/erac399)
Supplement: erac399_suppl_Supplementary_Figures_S1-S20 [file erac399_suppl_supplementary_figures_s1-s20.pdf]

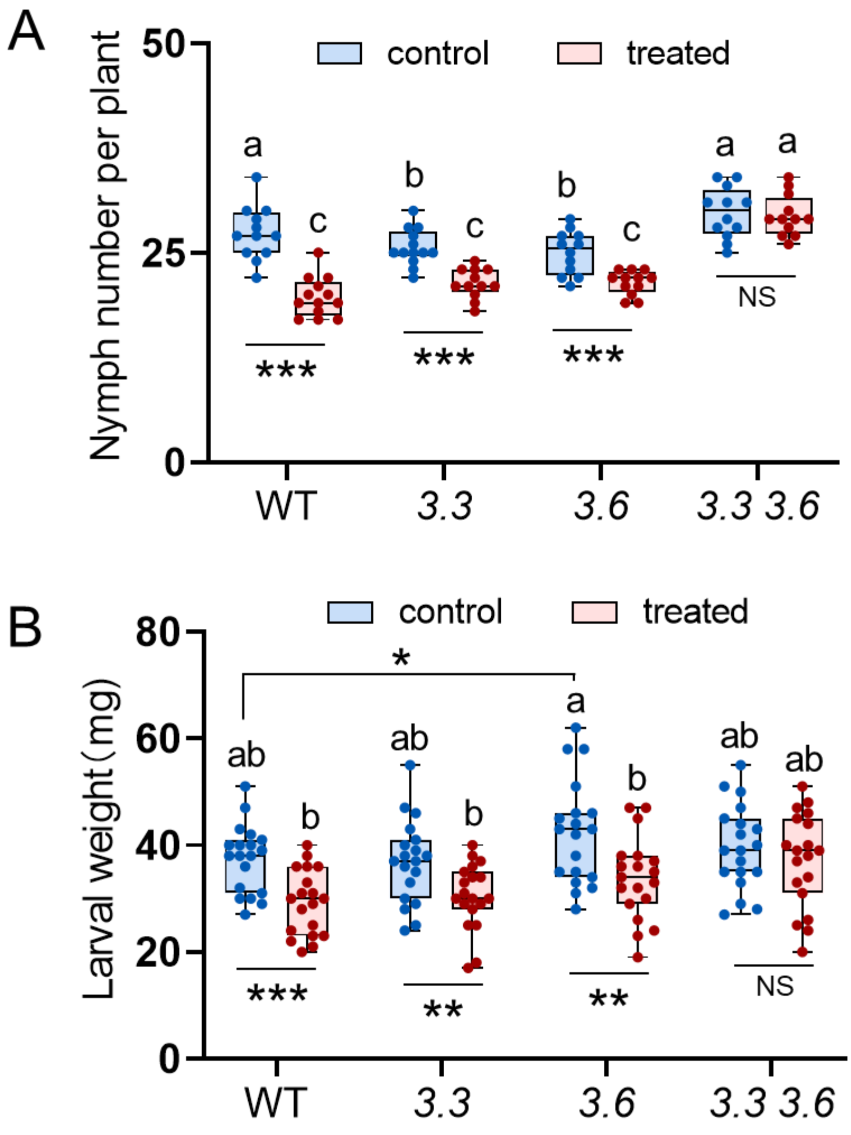

Fig. S1. The systemic resistance to GPA and CLW in WT plants, *glr3.3* and *glr3.6* single mutants, and *glr3.3 glr3.6* double mutants.

(A) GPA feeding-induced resistance to GPA in systemic leaf of WT, *glr3.3* (*3.3*), *glr3.6* (*3.6*), and *glr3.3 glr3.6* (*3.3 3.6*) plants. The leaf 8 (L8) was infested with 50 GPAs (treated) or mocked treated (no GPAs; control); after three days, the systemic leaf 13 (L13) was infested with one adult GPA, and the number of its offspring was recorded every day for two weeks (n = 12). **(B)** Simulated CLW feeding-induced systemic resistance to CLW. The leaf (L8) was treated with W+OS (treated) or untreated (control), and after two days, two freshly hatched CLW larvae were placed on the systemic leaf L13; one day later, the biomasses of CLW larvae were recorded (n = 19). In each box plot, the center line locates the median, the box

encompasses the upper and lower quartiles, each dot in the box plots indicates a specific sample, the error bars show the maximum and minimum of the distributions. Two-tailed Student's; * $P < 0.05$, ** $P < 0.01$, *** $P < 0.001$, NS = not significant. Different letters indicate significant difference (two-way ANOVA followed by Tukey's multiple comparisons test, $P < 0.05$).

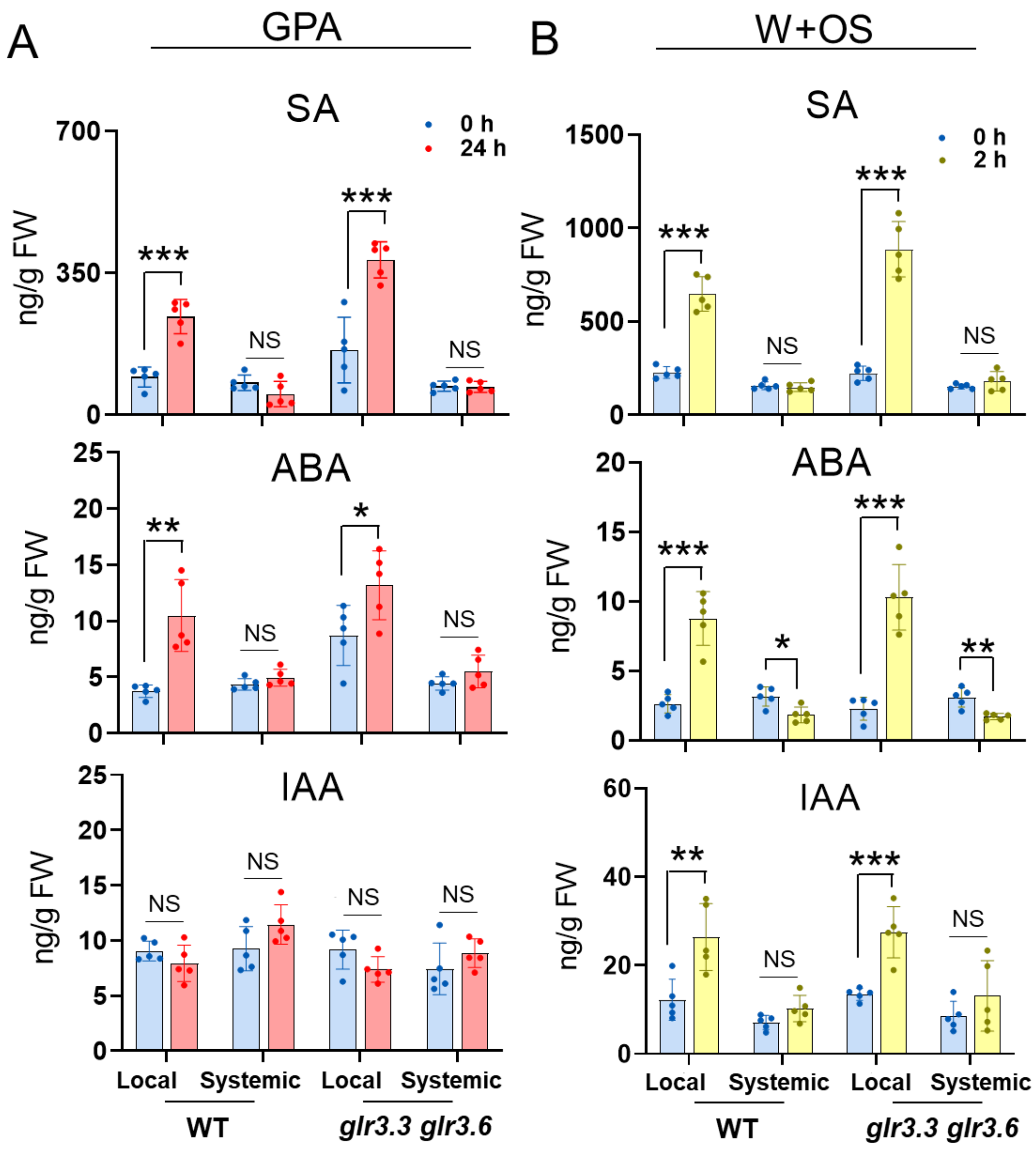

Fig. S2. Quantification of SA, ABA, and IAA levels in WT and *glr3.3 glr3.6* plants in response to GPA and W+OS treatment.

(A, B) SA, ABA, and IAA concentrations in local and systemic leaves of WT and *glr3.3 glr3.6* plants in response to GPA feeding for 24 h (0 h = control) **(A)** or 2 h after W+OS treatment (0 h = control) **(B)**. FW, fresh weight. Values are the means ± SD. Two-tailed Student's; * $P < 0.05$, ** $P < 0.01$, *** $P < 0.001$, NS = not significant. n = 5.

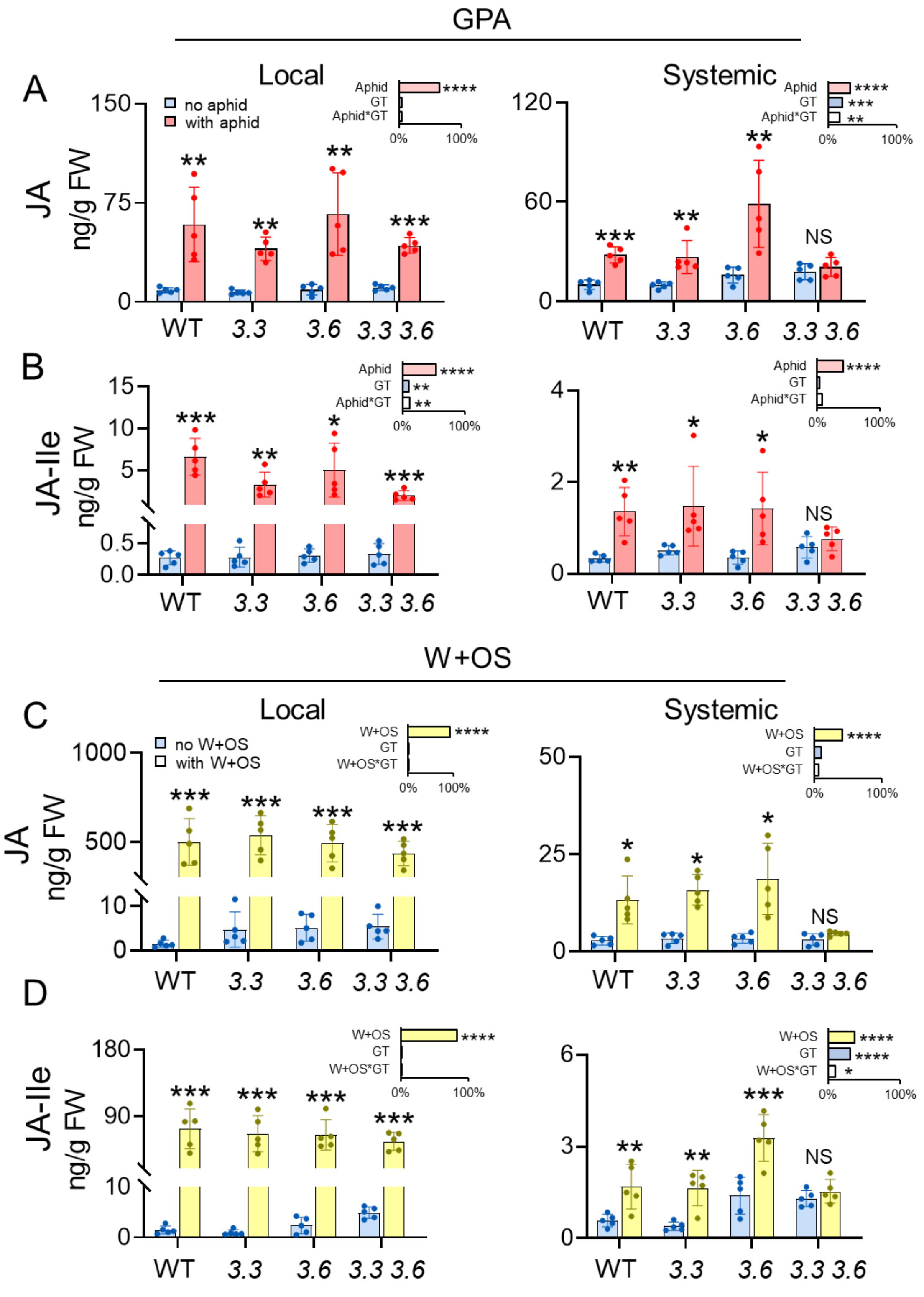

Fig. S3. Quantification of JA and JA-Ile level in WT plants, *glr3.3* and *glr3.6* single mutants, and *glr3.3 glr3.6* double mutants in response to GPA and W+OS treatment.

(A) JA and **(B)** JA-Ile concentrations in local and systemic leaves of WT, *glr3.3* (*3.3*), *glr3.6*

(*3.6*), and *glr3.3 glr3.6* (*3.3 3.6*) plants before (no aphid) or after GPA feeding 24 h (with aphid). **(C)** JA and **(D)** JA-Ile concentrations in local and systemic leaves of WT, *glr3.3*, *glr3.6*, and *glr3.3 glr3.6* plants before (no W+OS) or 2h after W+OS treatment (with W+OS). FW, fresh weight. Values are the means ± SD. Two-tailed Student's; * $P < 0.05$, ** $P < 0.01$, *** $P < 0.001$, NS = not significant. n = 5. The insets show the contribution (%) of factors, which were tested with two-way ANOVA considering genotype (GT) and aphid feeding (aphid) or W+OS treatment as factors, followed by Tukey's multiple comparisons test. In insets, * $P < 0.05$, ** $P < 0.01$, *** $P < 0.001$, **** $P < 0.0001$.

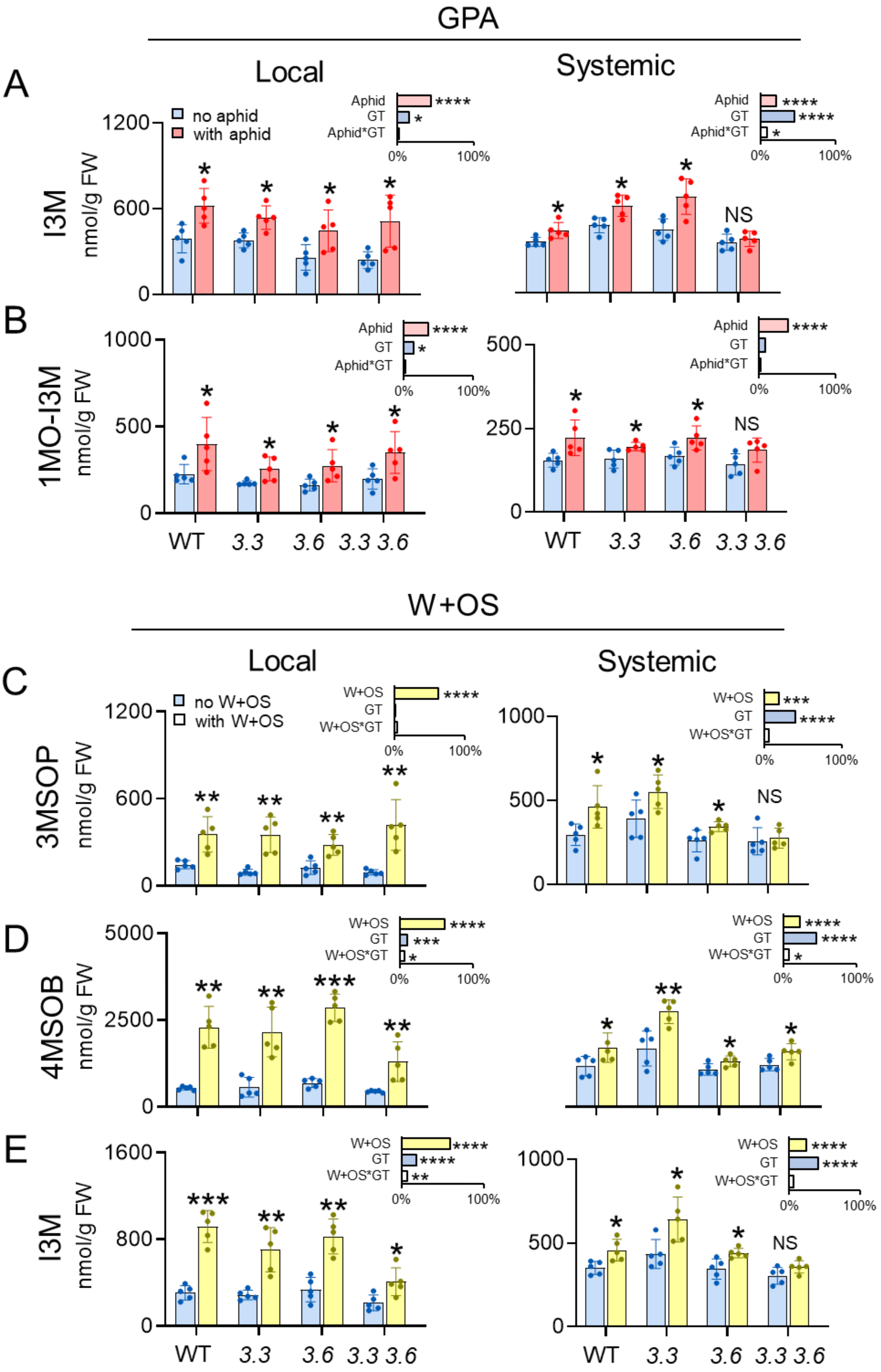
GPA
A
Local
Systemic
I3M
nmol/g FW
no aphid
with aphid
Aphid
GT
Aphid*GT
****
*
0%
100%
NS
B
1MO-I3M
WT
3.3
3.6
3.3 3.6
W+OS
C
3MSOP
no W+OS
with W+OS
W+OS*GT
D
4MSOB
E
I3M

Fig. S4. Quantification of GSs in WT, *glr3.3* and *glr3.6* single mutants, and *glr3.3 glr3.6* double mutants in response to GPA and W+OS treatment.

(A, B) The contents of I3M **(A)** and 1MO-I3M **(B)** in local and systemic leaves of WT, *glr3.3* (*3.3*), *glr3.6* (*3.6*), and *glr3.3 glr3.6* (*3.3 3.6*) plants in response to GPA feeding for 3 days (with aphid) or under control conditions (no aphid). **(C to E)** The contents of 3MSOP **(C)**, 4MSOB **(D)**, and I3M **(E)** in local and systemic leaves of WT, *glr3.3*, *glr3.6*, and *glr3.3 glr3.6* plants in response to W+OS treatment for 2 days (with W+OS) or under control conditions (no W+OS). FW, fresh weight. Values are the means ± SD. Two-tailed Student's t test (**P* < 0.05, ***P* < 0.01, ****P* < 0.001, NS = not significant. n = 5). The insets show the contribution (%) of factors, which were tested with two-way ANOVA considering genotype (GT) and aphid feeding (aphid) or W+OS treatment as factors, followed by Tukey's multiple comparisons test. In insets, * *P* < 0.05, ** *P* < 0.01, *** *P* < 0.001, **** *P* < 0.0001.

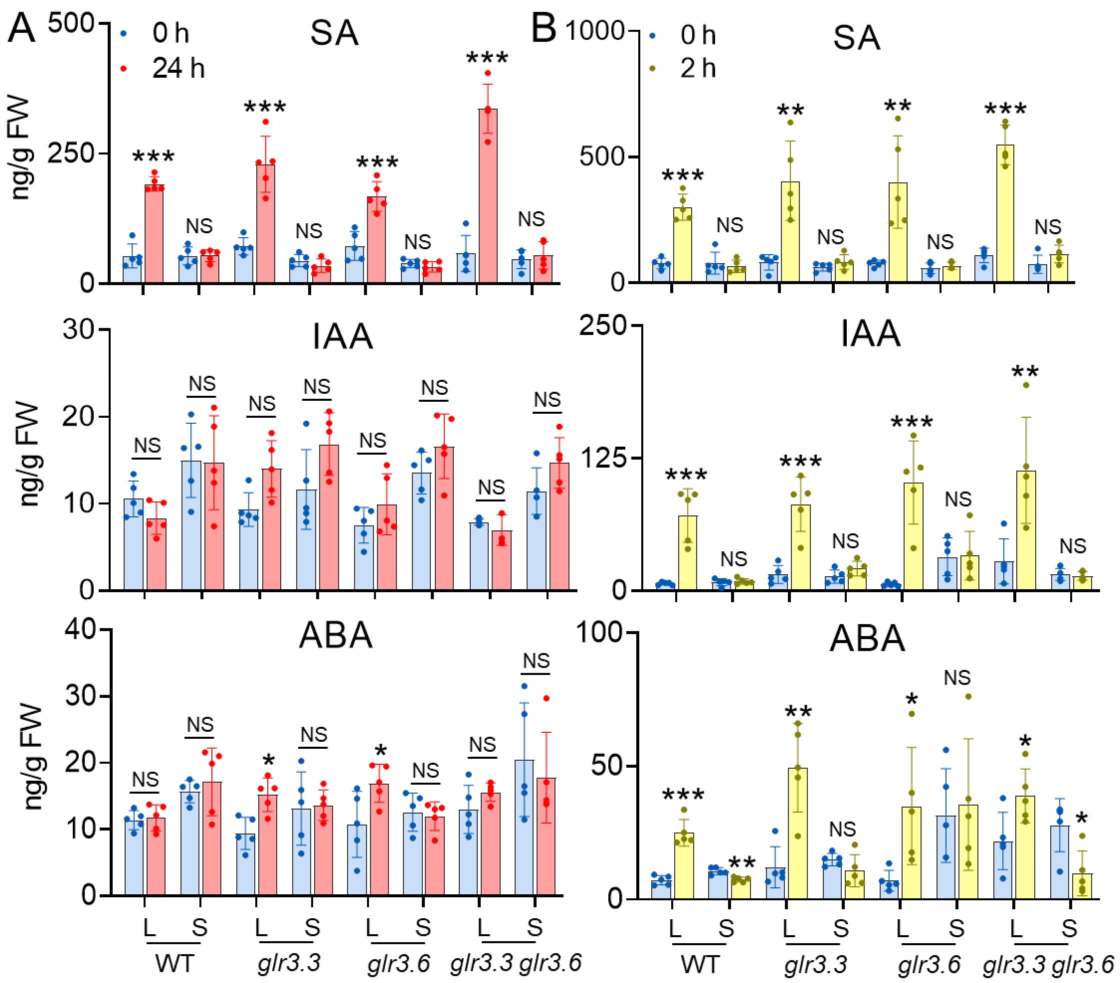

Fig. S5. Quantification of SA, ABA, and IAA levels in WT plants, *glr3.3* and *glr3.6* single mutants, and *glr3.3 glr3.6* double mutants in response to GPA and W+OS treatment.

(A, B) SA, ABA, and IAA concentrations in local (L) and systemic (S) leaves of WT, *glr3.3*, *glr3.6*, and *glr3.3 glr3.6* plants after GPA feeding for 24 h (0 h = control) **(A)** or 2 h W+OS treatment (0 h = control) **(B)**. FW, fresh weight. Values are the means ± SD. Two-tailed Student's; * $P < 0.05$, ** $P < 0.01$, *** $P < 0.001$, NS = not significant. n = 5.

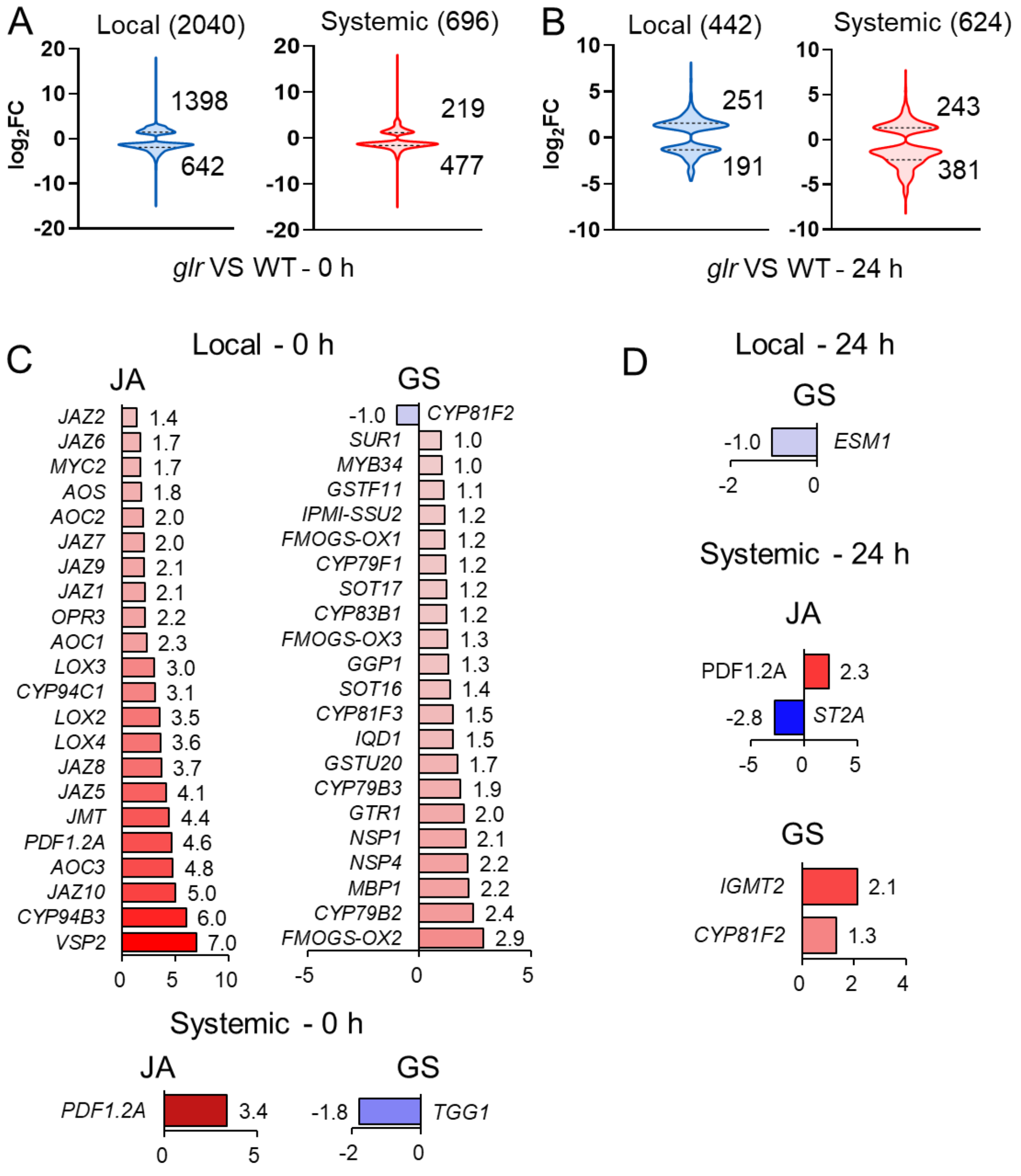

Fig. S6. DEGs in local and systemic leaves of *glr3.3 glr3.6* mutants compared with WT under control conditions and after GPA feeding for 24 h.

(A, B) Violin plots indicating the numbers and relatively expression of DEGs (log_2FC) in local and systemic leaves of *glr3.3 glr3.6* (*glr*) mutants compared with WT plants under control conditions (0 h) **(A)** and after 24 h of GPA feeding **(B)**. Dashed lines indicate quartiles. The numbers in the brackets indicate the total numbers of DEGs. **(C, D)** Heatmaps indicating the relative expression levels (log_2FC) of JA- and GS- related genes in local and systemic leaves between *glr3.3 glr3.6* mutants and WT plants under control conditions (0 h) **(C)** and 24 h after GPA feeding **(D)**. Complete data can be found in Table S1.

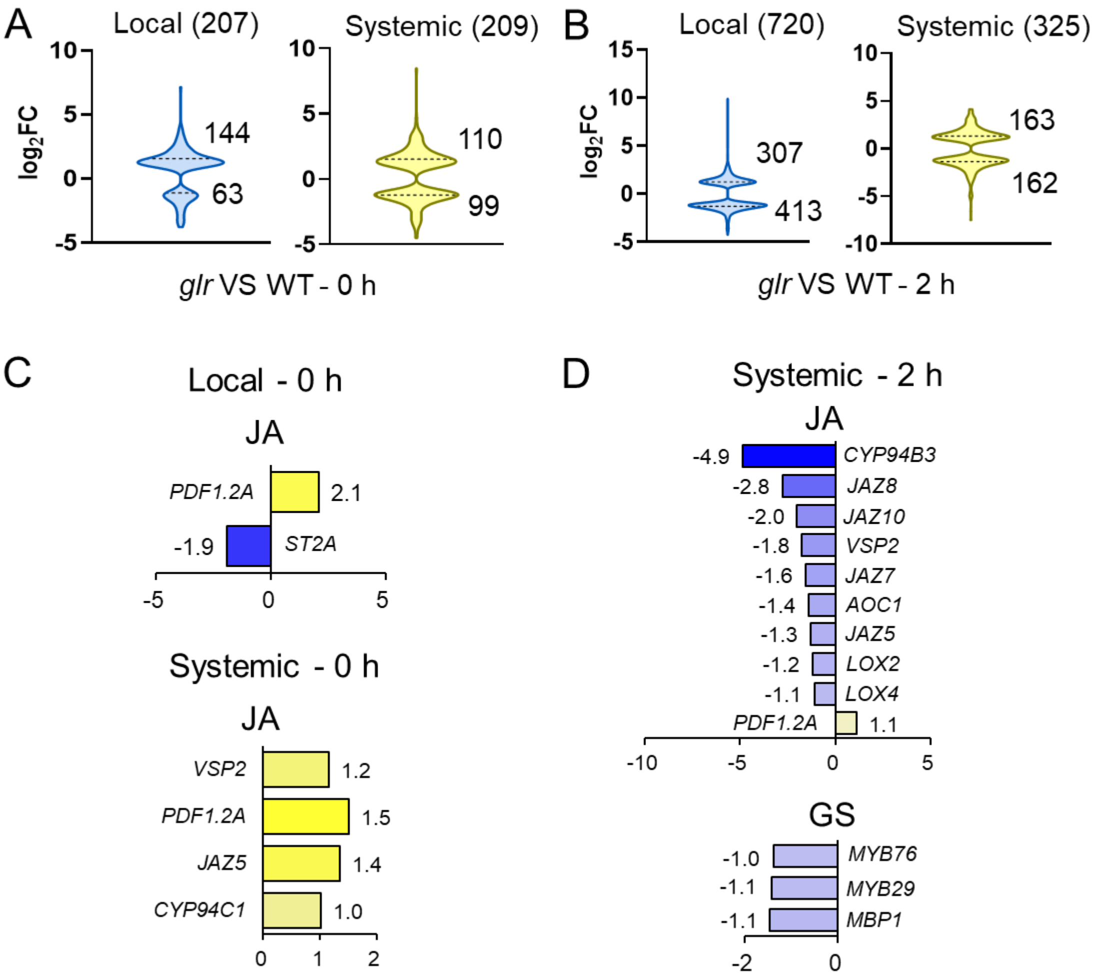

Fig. S7. DEGs in local and systemic leaves of *glr3.3 glr3.6* mutants compared with WT plants under control conditions and 2 h after W+OS treatment.

(A, B) Violin plots indicating the numbers and relatively expression of DEGs (log_2FC) in local and systemic leaves of *glr3.3 glr3.6* (*glr*) mutants compared with WT plants under control conditions (0 h) **(A)** and 2 h after W+OS treatment **(B)**. Dashed lines indicate quartiles. The numbers in the brackets indicate the total numbers of DEGs. **(C, D)** Heatmaps indicating the relative expression levels (log_2FC) of JA- and GS- related genes between *glr3.3 glr3.6* mutants and WT plants in local and systemic leaves under control conditions (0 h) **(C)** and 2 h after W+OS treatment **(D)**. Complete data can be found in Table S1.

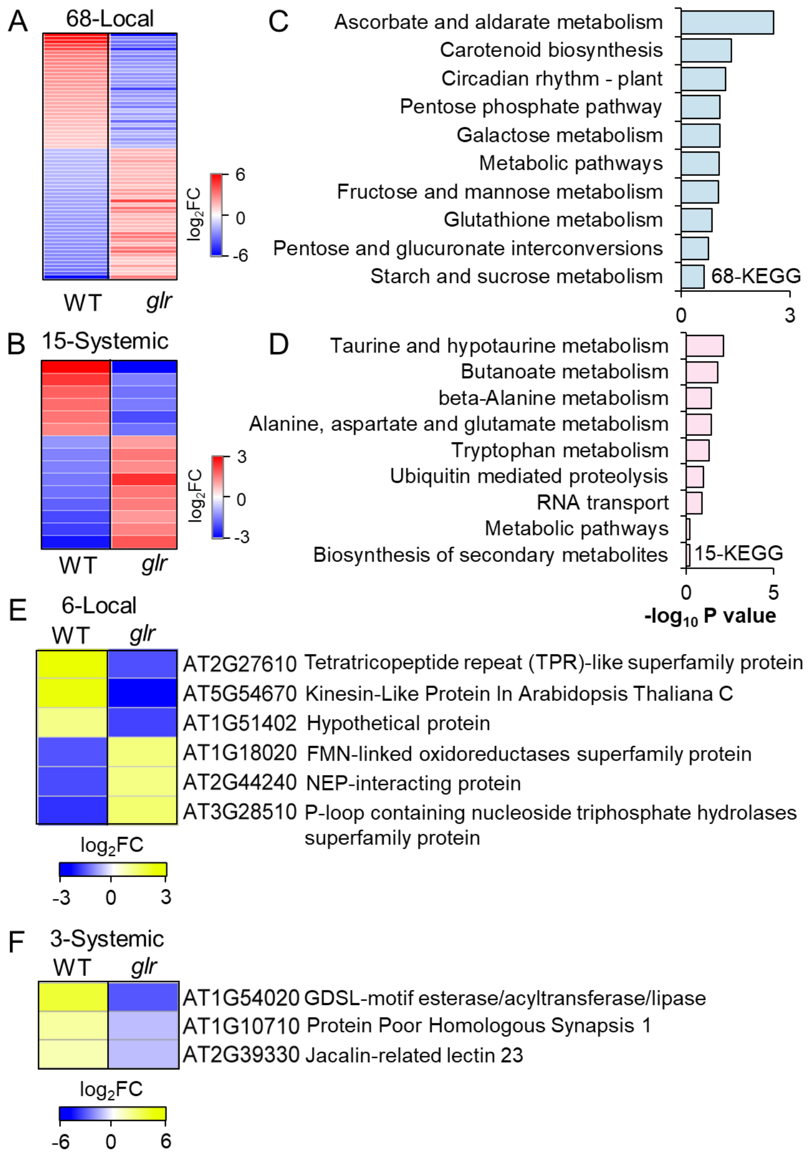

Fig. S8. Common DEGs identified in WT and *glr3.3 glr3.6* plants which had opposite directions of regulation.

(A, B) Heatmaps indicating the relative expression of DEGs, which were common between GPA feeding-induced WT and *glr3.3 glr3.6* plants but exhibited opposite directions of regulation, in local **(A)** and systemic **(B)** leaves. **(C, D)** KEGG pathway analysis of the 66 and 15 DEGs respectively shown in **(A)** and **(B)**. **(E, F)** Heatmaps indicating the relative expression of DEGs, which were common between W+OS-induced WT and *glr3.3 glr3.6* plants but exhibited opposite directions of regulation, in local **(E)** and systemic **(F)** leaves. Complete data can be found in Table S2 and S3.

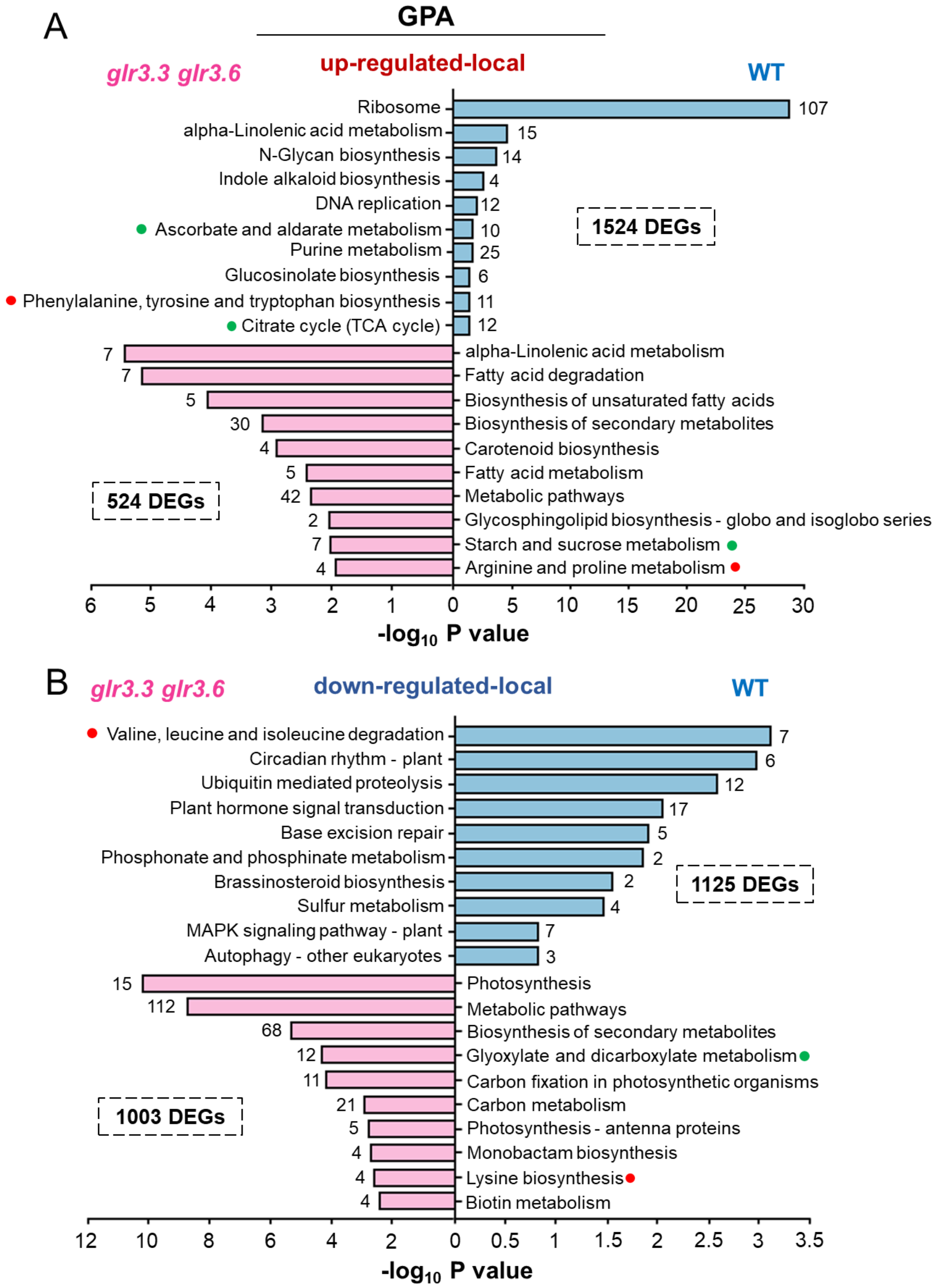

Fig. S9. KEGG enrichment analysis on the DEGs in local leaves of WT and *glr3.3 glr3.6* plants in response to GPA Infestation.

(A, B) Top 10 KEGG pathways enriched from the up- **(A)** and down- **(B)** regulated DEGs uniquely identified in local leaves of WT and *glr3.3 glr3.6* plants after GPA feeding for 24 h. Red and green dots highlight the pathways of amino acids and carbohydrate metabolism, respectively. Complete data can be found in Table S4.

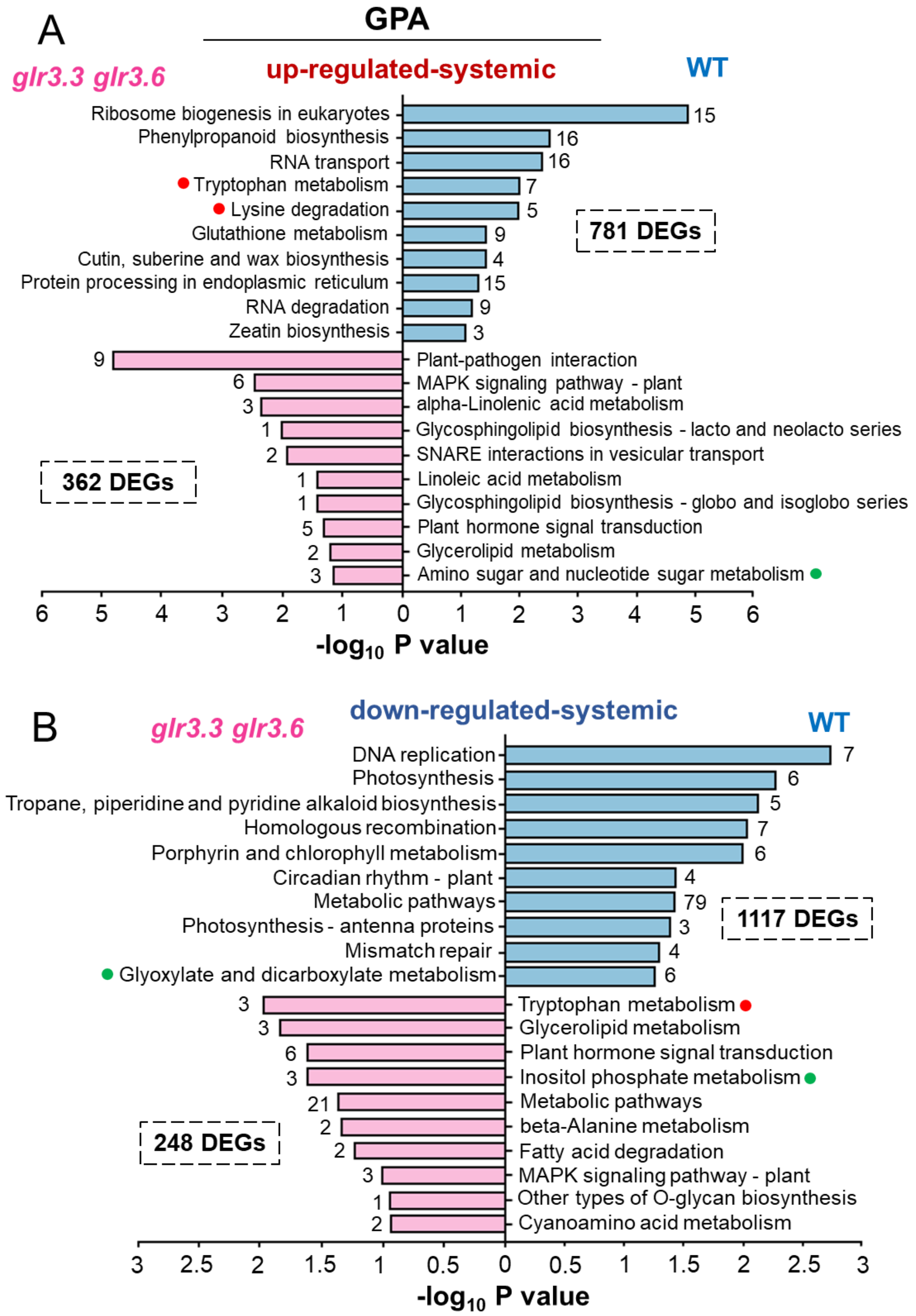

Fig. S10. KEGG enrichment analysis on DEGs in systemic leaves of WT and *glr3.3 glr3.6* plants in response to GPA Infestation.

(A, B) Top 10 KEGG pathways enriched from the up- **(A)** and down- **(B)** regulated DEGs uniquely identified in systemic leaves of WT and *glr3.3 glr3.6* plants after GPA feeding local leaves for 24 h. Red and green dots highlight the pathways of amino acids and carbohydrate metabolism, respectively. Complete data can be found in Table S4.

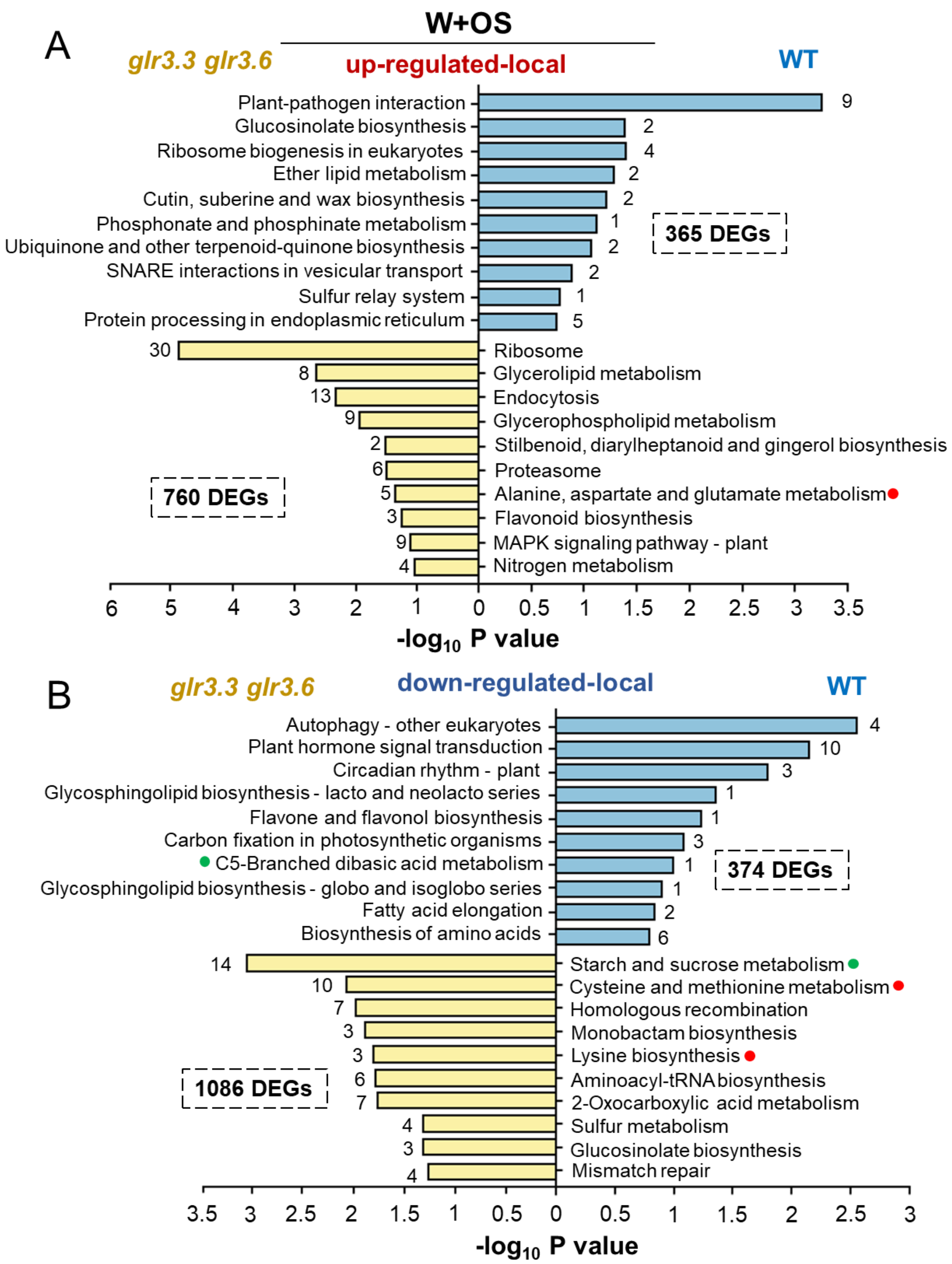

Fig. S11. KEGG enrichment analysis on DEGs in local leaves of WT and *glr3.3 glr3.6* plants in response to W+OS treatment.

(A, B) Top 10 KEGG pathways enriched from the up- **(A)** and down- **(B)** regulated DEGs uniquely identified in local leaves of WT and *glr3.3 glr3.6* plants after W+OS treatment for 2 h. Red and green dots highlight the pathways of amino acids and carbohydrate metabolism, respectively. Complete data can be found in Table S5.

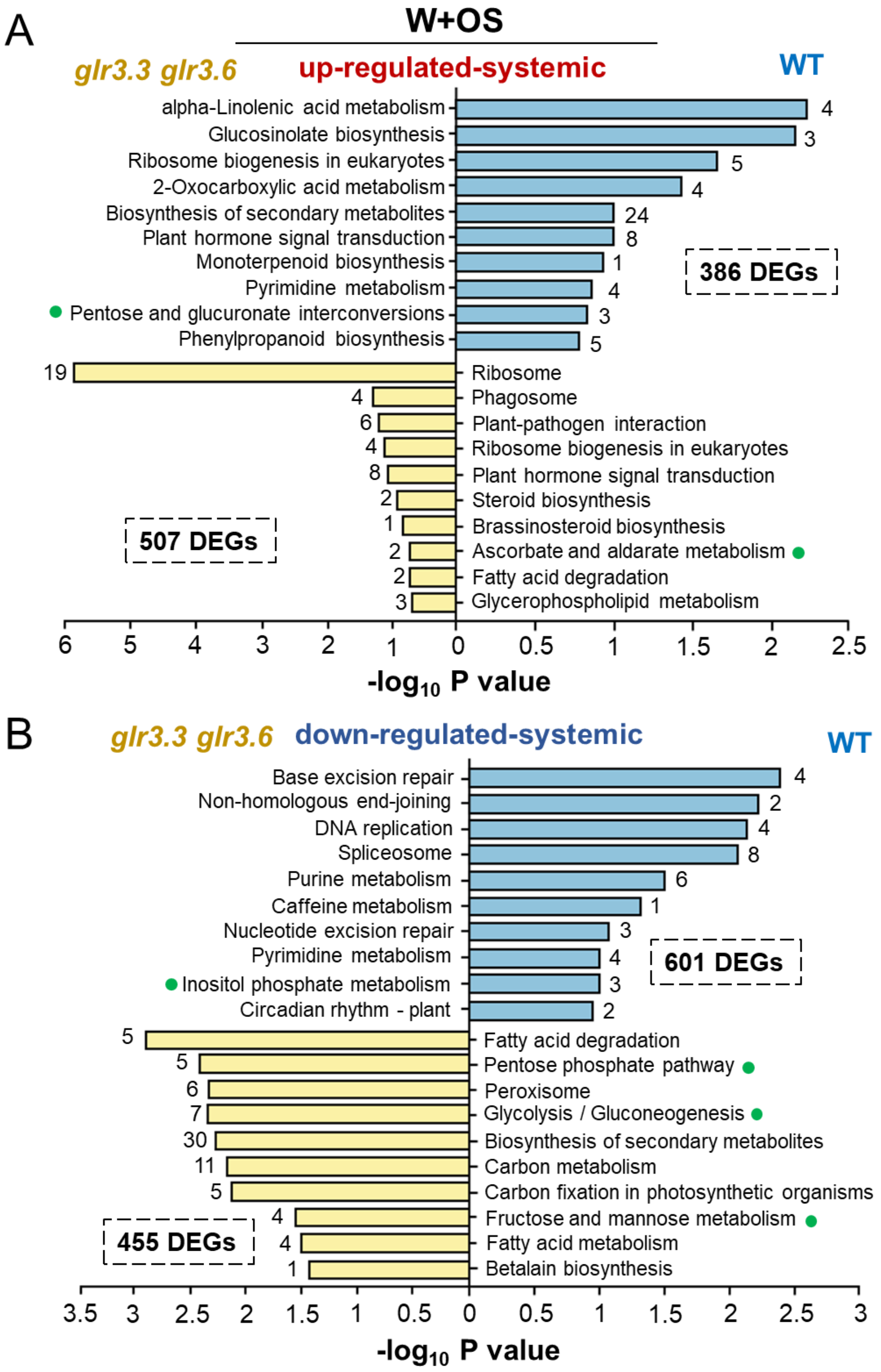

Fig. S12. KEGG enrichment analysis on DEGs in systemic leaves of WT and *glr3.3 glr3.6* plants in response to W+OS treatment.

(A, B) Top 10 KEGG pathways enriched from the up- **(A)** and down- **(B)** regulated DEGs uniquely identified in systemic leaves of WT and *glr3.3 glr3.6* plants after W+OS treatment for 2 h. Red and green dots highlight the pathways of amino acids and carbohydrate metabolism, respectively. Complete data can be found in Table S5.

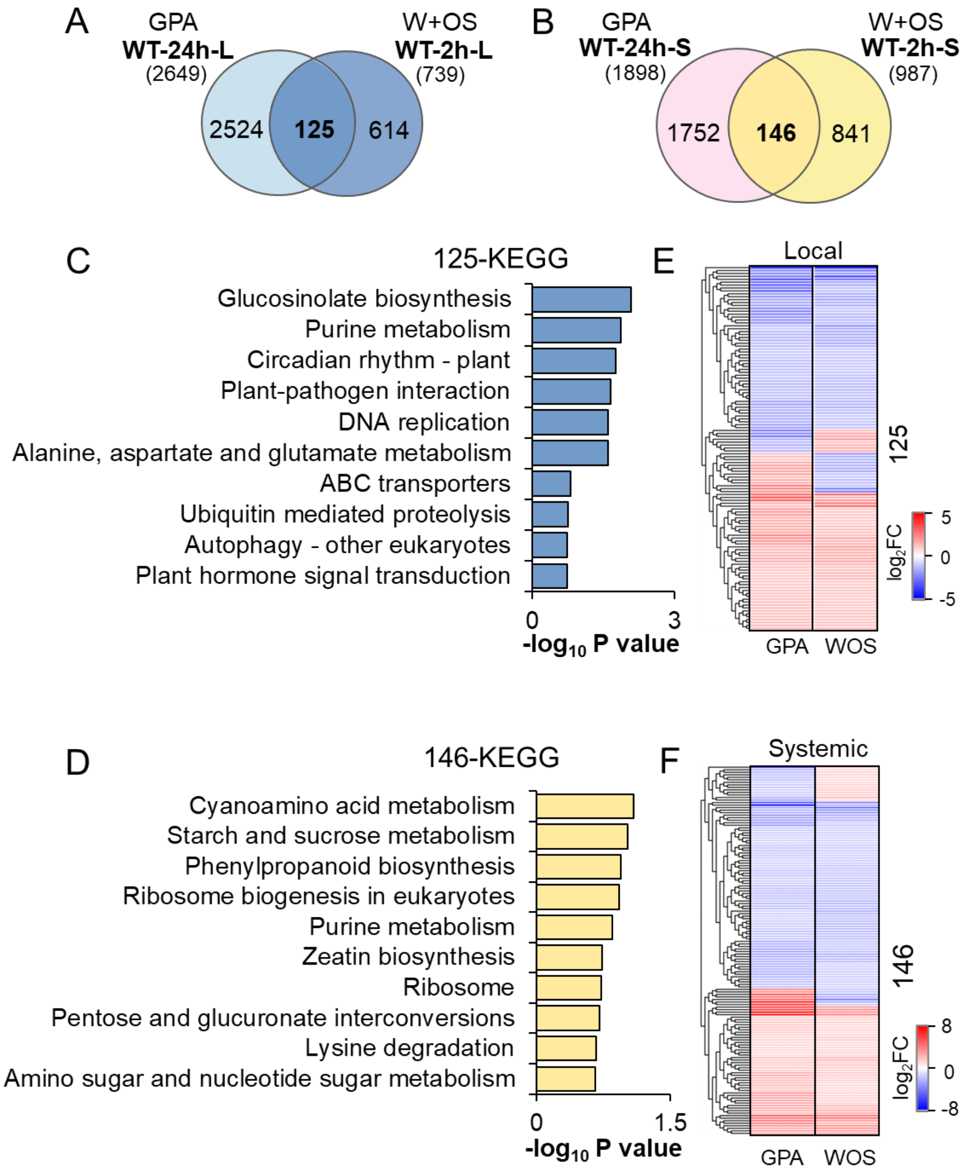

Fig. S13. The uniquely regulated DEGs in local and systemic leaves of WT plants in response to GPA feeding and W+OS treatment.

WT and *glr3.3 glr3.6* (*glr*) plants were treated with GPA feeding for 24 h or W+OS for 2 h, and local (L) and systemic (S) leaves were harvested for RNA-seq analysis. **(A, B)** Venn diagram analysis on the uniquely regulated DEGs in local **(A)** and systemic **(B)** leaves of WT plants between GPA feeding and W+OS treatment. The numbers in the brackets indicate the total numbers of uniquely regulated DEGs. **(C)** and **(D)** indicate the top 10 enriched pathways of the 125 and 146 common genes. **(E, F)** Heatmaps indicating the relative expression of the 125 **(E)** and 146 **(F)** common genes. Complete data can be found in Table S7.

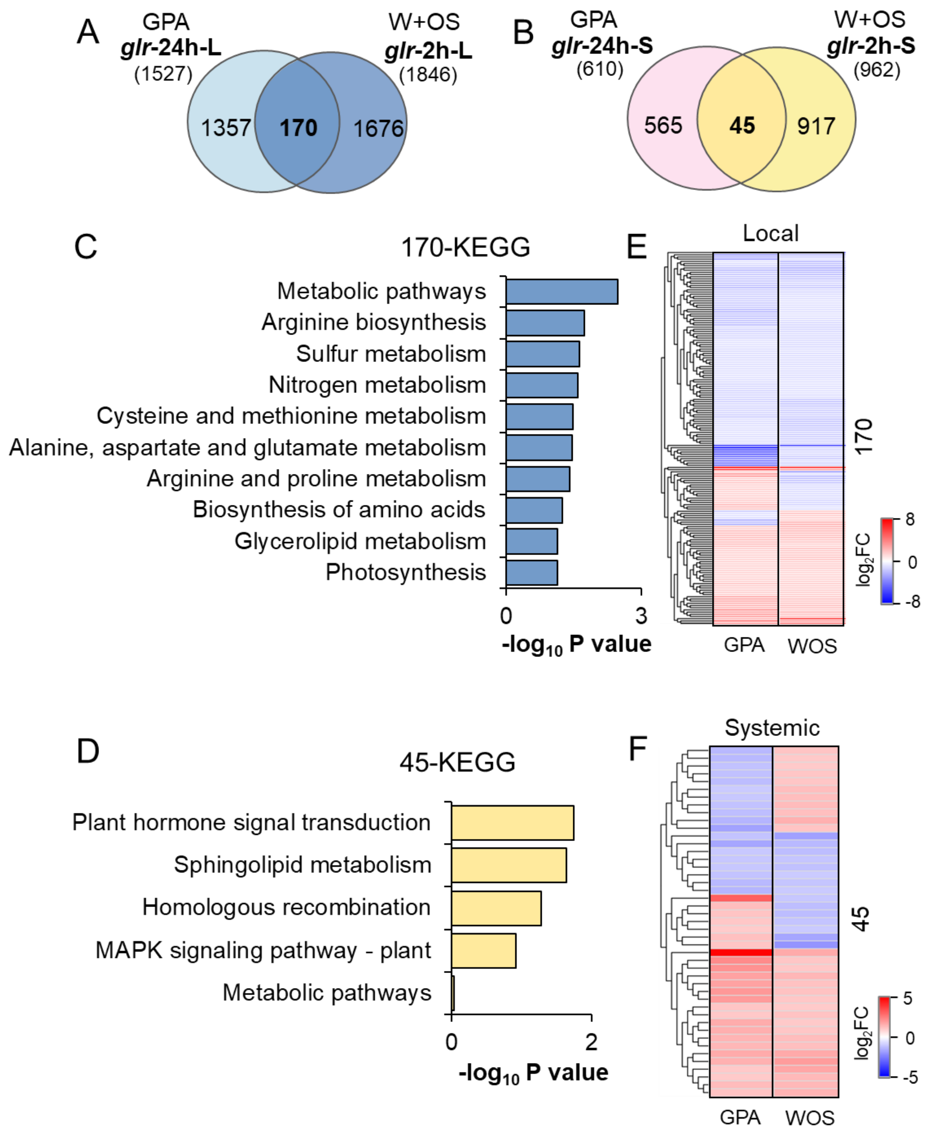

Fig. S14. Uniquely regulated DEGs in local and systemic leaves of *glr3.3 glr3.6* mutants in response to GPA and W+OS treatment.

WT and *glr3.3 glr3.6* (*glr*) plants were treated with GPA feeding for 24 h or W+OS for 2 h, and local (L) and systemic (S) leaves were harvested for RNA-seq analysis. **(A, B)** Venn diagram analysis on the uniquely regulated DEGs in local **(A)** and systemic **(B)** leaves of *glr3.3 glr3.6* between GPA feeding and W+OS treatment. The numbers in the brackets indicate the total numbers of uniquely regulated DEGs. **(C)** and **(D)** indicate the top 10 enriched pathways of the 170 and 45 common genes. **(E, F)** Heatmaps indicating the relative expression of the 170 **(E)** and 45 **(F)** common genes. Complete data can be found in Table S7.

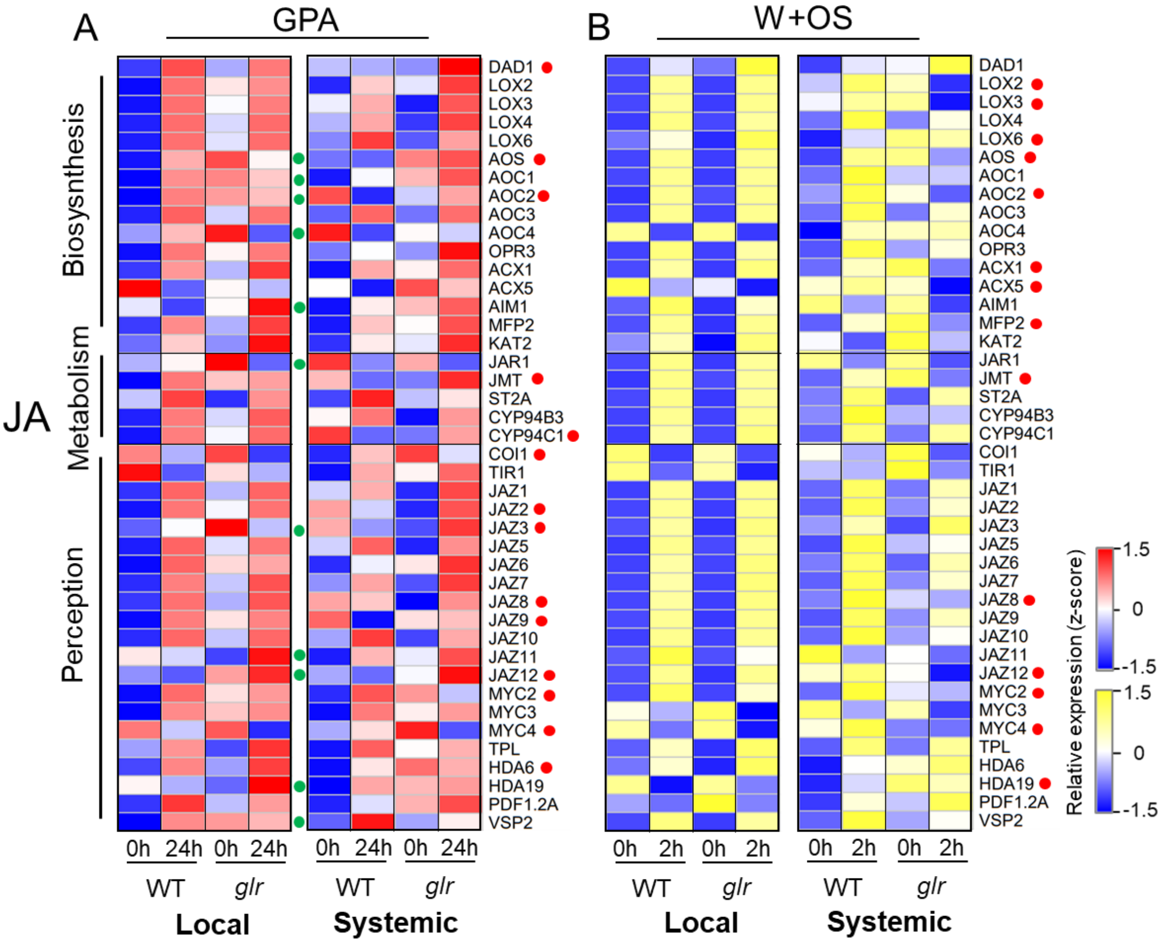

Fig. S15. Relative transcript levels of genes involved in JA biosynthesis, metabolism, and perception.

(A, B) Heatmaps indicating the relative transcript levels of JA metabolism and signaling genes in local and systemic leaves of WT and *glr3.3 glr3.6* (*glr*) plants in response to GPA infestation for 24 h **(A)** or W+OS treatment for 2 h **(B)**. Green and red dots depict genes whose levels were differentially regulated between *glr3.3 glr3.6* and WT plants in local or systemic leaves, respectively. Relative expression levels (VSTs) of genes were first $\log_2$ transformed and then normalized using the Z-score algorithm. Each block represents the mean of 3 biological replicates. Gene names and transcript levels are listed in Table S8 and S9.

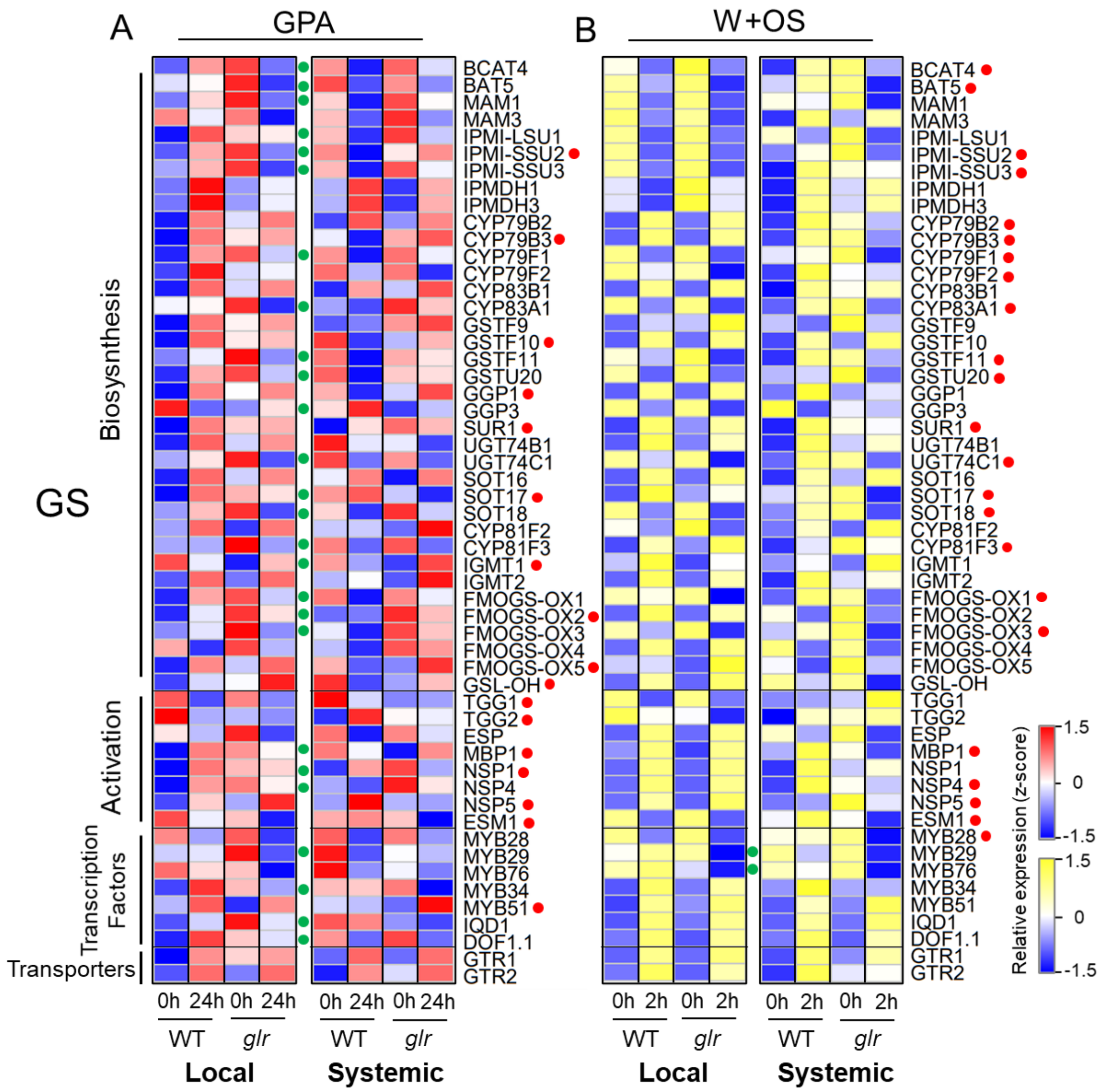

Fig. S16. Relative transcripts level of genes involved in GS biosynthesis, activation, regulation, and transport.

(A, B) Heatmaps indicating the relative transcript levels of genes involved in GS biosynthesis, activation, regulation, and transport in local and systemic leaves of WT and *glr3.3 glr3.6* (*glr*) plants after GPA infestation for 24 h **(A)** or W+OS treatment for 2 h **(B)**. Green and red dots depict genes whose levels were differentially regulated between *glr3.3 glr3.6* and WT plants in local or systemic leaves, respectively. Relative expression levels (VSTs) of genes were first $\log_2$ transformed and then normalized using the Z-score algorithm. Each block represents the mean of 3 biological replicates. Gene names and transcript levels are listed in Table S8 and S9.

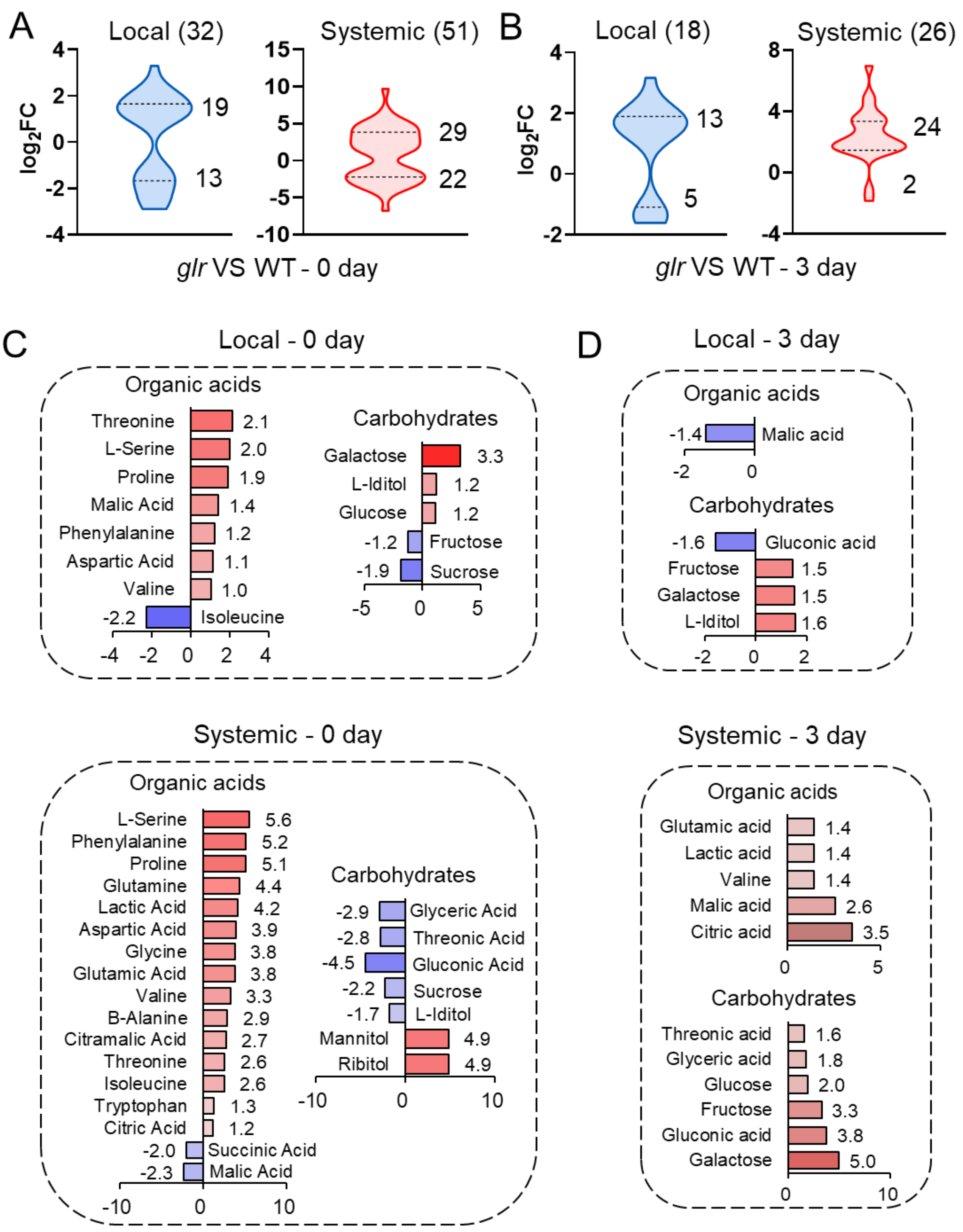

Fig. S17. DPMs in local and systemic leaves of *glr3.3 glr3.6* mutants compared with WT plants under control conditions and after GPA feeding for 3 days.

(A, B) Violin plots indicating the numbers and relatively expression of DPMs (log_2FC) in local and systemic leaves of *glr3.3 glr3.6* (*glr*) mutants compared with WT plants under control conditions (0 day) **(A)** and after GPA feeding for 3 days **(B)**. Dashes lines indicate quartiles.

The numbers in the brackets indicate the total numbers of DPMs. **(C, D)** Heatmaps indicating the relative concentrations (log_2FC) of organic acids and carbohydrates between *glr3.3 glr3.6* mutants and WT plants in local and systemic leaves under control conditions (0 day) **(C)** and after GPA feeding for 3 days **(D)**. Complete data can be found in Table S10.

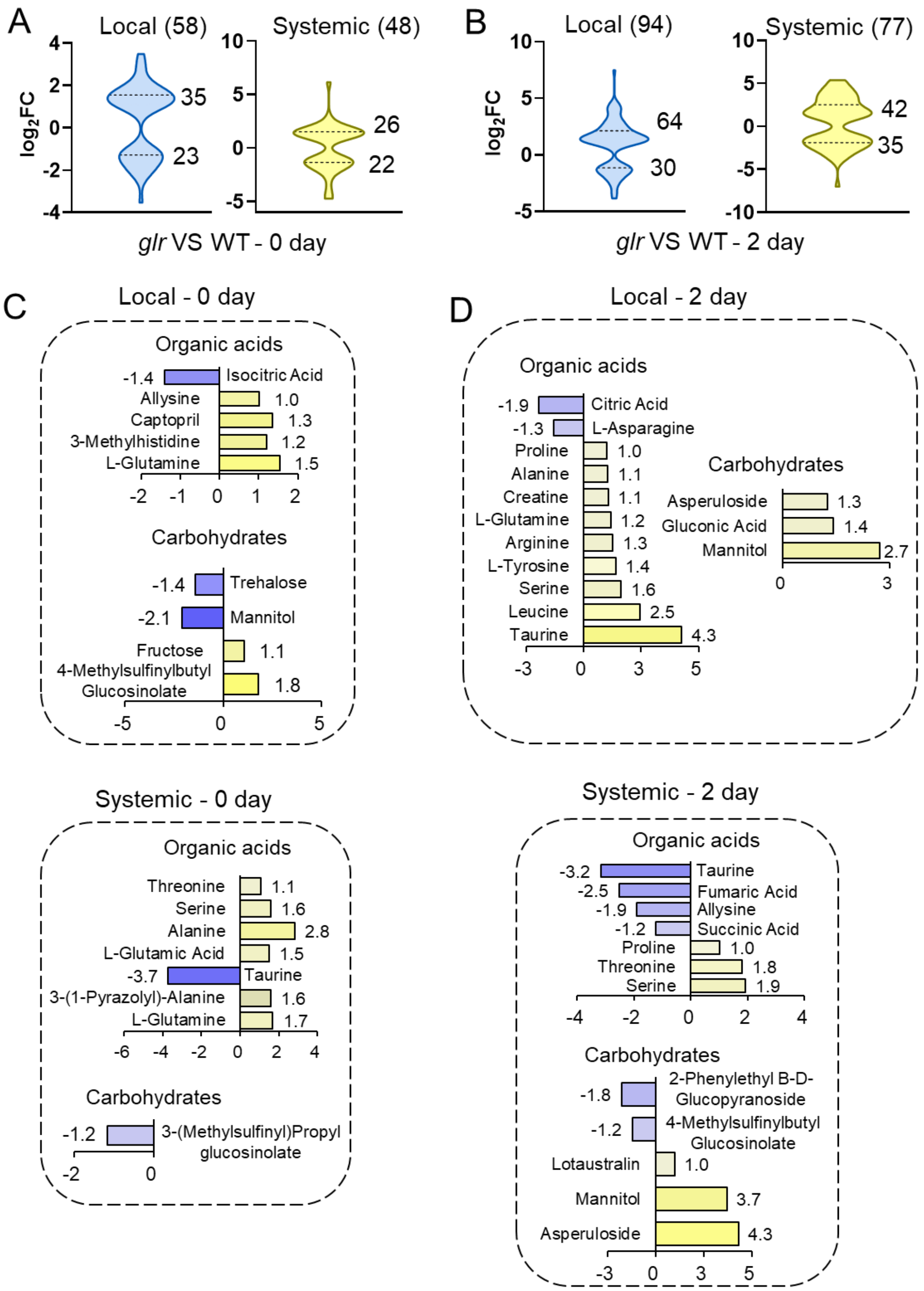

Fig. S18. DPMs in local and systemic leaves of *glr3.3 glr3.6* mutants compared with WT plants under control conditions and after W+OS treatment for 2 days.

(A, B) Violin plots indicating the numbers and relatively expression of DPMs (log$_2$FC) in local and systemic leaves of *glr3.3 glr3.6* (*glr*) mutants compared with WT plants under control

conditions (0 day) **(A)** and after W+OS treatment for 2 days **(B)**. Dashed lines indicate quartiles. The numbers in the brackets indicate the total numbers of DPMs. **(C, D)** Heatmaps indicating the relative concentrations (log_2FC) of organic acids and carbohydrates between *glr3.3 glr3.6* mutants and WT plants in local and systemic leaves under control conditions (0 day) **(C)** and 2 days after W+OS treatment **(D)**. Complete data can be found in Table S10.

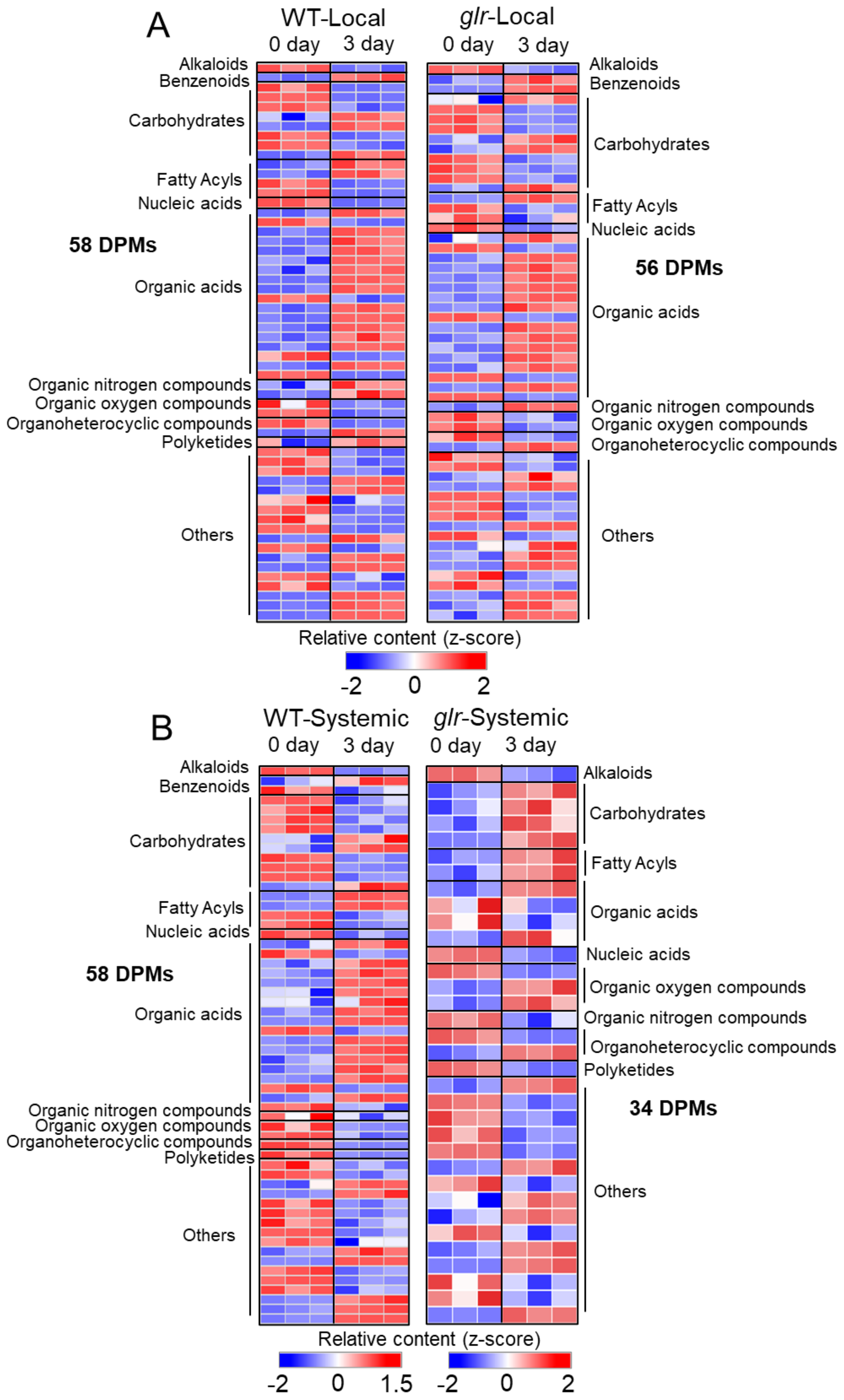
A
WT-Local
glr-Local
0 day
3 day
0 day
3 day
Alkaloids
Benzenoids
Carbohydrates
Fatty Acyls
Nucleic acids
58 DPMs
Organic acids
Organic nitrogen compounds
Organic oxygen compounds
Organoheterocyclic compounds
Polyketides
Others
56 DPMs
Relative content (z-score)
-2
0
2
B
WT-Systemic
glr-Systemic
0 day
3 day
0 day
3 day
Alkaloids
Benzenoids
Carbohydrates
Fatty Acyls
Nucleic acids
58 DPMs
Organic acids
Organic nitrogen compounds
Organic oxygen compounds
Organoheterocyclic compounds
Polyketides
Others
34 DPMs
Relative content (z-score)
-2
0
1.5
-2
0
2

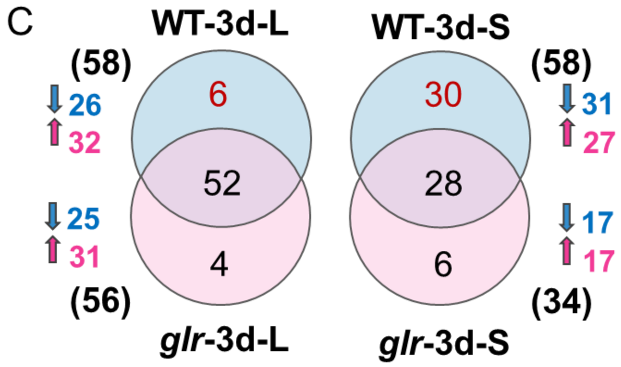

Fig. S19. Relative contents of differentially produced metabolites in the local and systemic leaves of WT and *glr3.3 glr3.6* plants under GPA infestation condition.

After GPA feeding on WT and *glr3.3 glr3.6* (*glr*) plants for three days, the local and systemic leaves were harvested for quantification of metabolites. **(A, B)** Heatmaps depicting the relative concentrations of DPMs in local **(A)** and systemic **(B)** leaves of WT and *glr3.3 glr3.6* plants. Relative contents of metabolites were first log_2 transformed and then normalized using the Z-score algorithm. Each block represents one biological replicate (n = 3). **(C)** Venn diagrams indicating the numbers of common and specific DPMs identified between WT and *glr3.3 glr3.6* (*glr*) plants in local (L) and systemic (S) leaves (shown as genotype-time of treatment-local/systemic, e.g., WT-3d-L). Complete data can be found in Table S11.

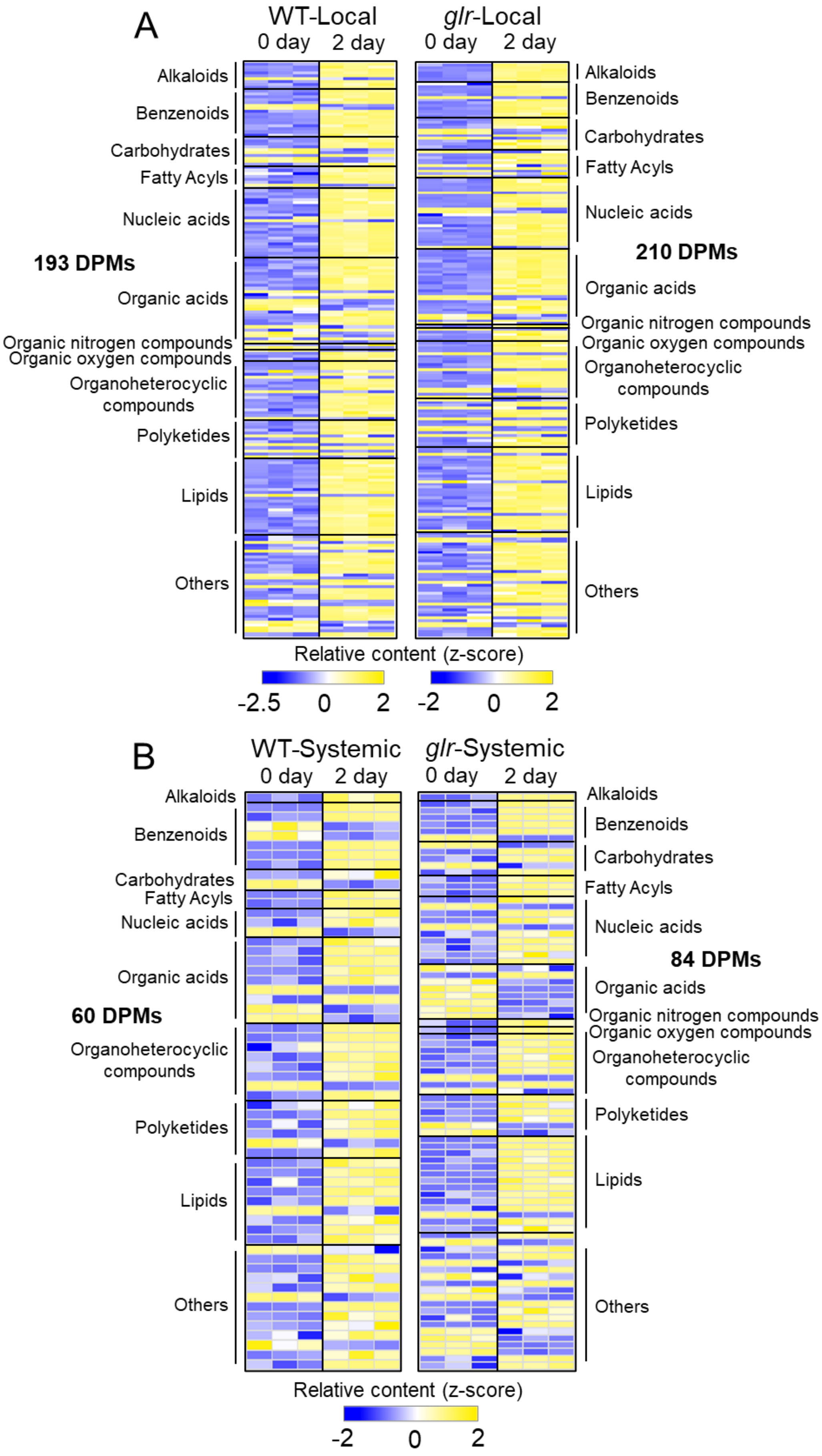
A
WT-Local
0 day
2 day
glr-Local
0 day
2 day
Alkaloids
Benzenoids
Carbohydrates
Fatty Acyls
Nucleic acids
193 DPMs
Organic acids
Organic nitrogen compounds
Organic oxygen compounds
Organoheterocyclic compounds
Polyketides
Lipids
Others
210 DPMs
Relative content (z-score)
-2.5
0
2
-2
0
2
B
WT-Systemic
0 day
2 day
glr-Systemic
0 day
2 day
Alkaloids
Benzenoids
Carbohydrates
Fatty Acyls
Nucleic acids
Organic acids
60 DPMs
Organoheterocyclic compounds
Polyketides
Lipids
Others
84 DPMs
Organic nitrogen compounds
Organic oxygen compounds
Relative content (z-score)
-2
0
2

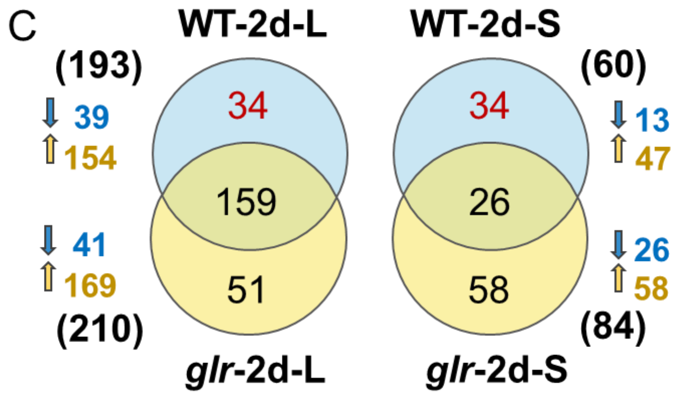

Fig. S20. Relative contents of differentially produced metabolites in the local and systemic leaves of WT and *glr3.3 glr3.6* plants under W+OS treatment condition.

After W+OS treatment on WT and *glr3.3 glr3.6* (*glr*) plants for two days, the local and systemic leaves were harvested for quantification of metabolites. **(A, B)** Heatmaps depicting the relative concentrations of DPMs in local **(A)** and systemic **(B)** leaves of WT and *glr3.3 glr3.6* plants. Relative contents of metabolites were first $\log_2$ transformed and then normalized using the Z-score algorithm. Each block represents one biological replicate (n = 3). **(C)** Venn diagrams indicating the numbers of common and specific DPMs between WT and *glr3.3 glr3.6* (*glr*) plants in local (L) and systemic (S) leaves (shown as genotype-time of treatment-local/systemic, e.g., WT-2d-L). Complete data can be found in Table S12.
