# Supplementary material for: The glutamate receptor-like 3.3 and 3.6 mediate systemic resistance to insect herbivores in Arabidopsis
Source: J Exp Bot. 2022 Oct 10;73(22):7611–27. doi: 10.1093/jxb/erac399 (PMC9730813; doi:10.1093/jxb/erac399)
Supplement: erac399_suppl_Supplementary_Videos_S1-S4 [file erac399_suppl_supplementary_videos_s1-s4.pptx]

## Slide 1
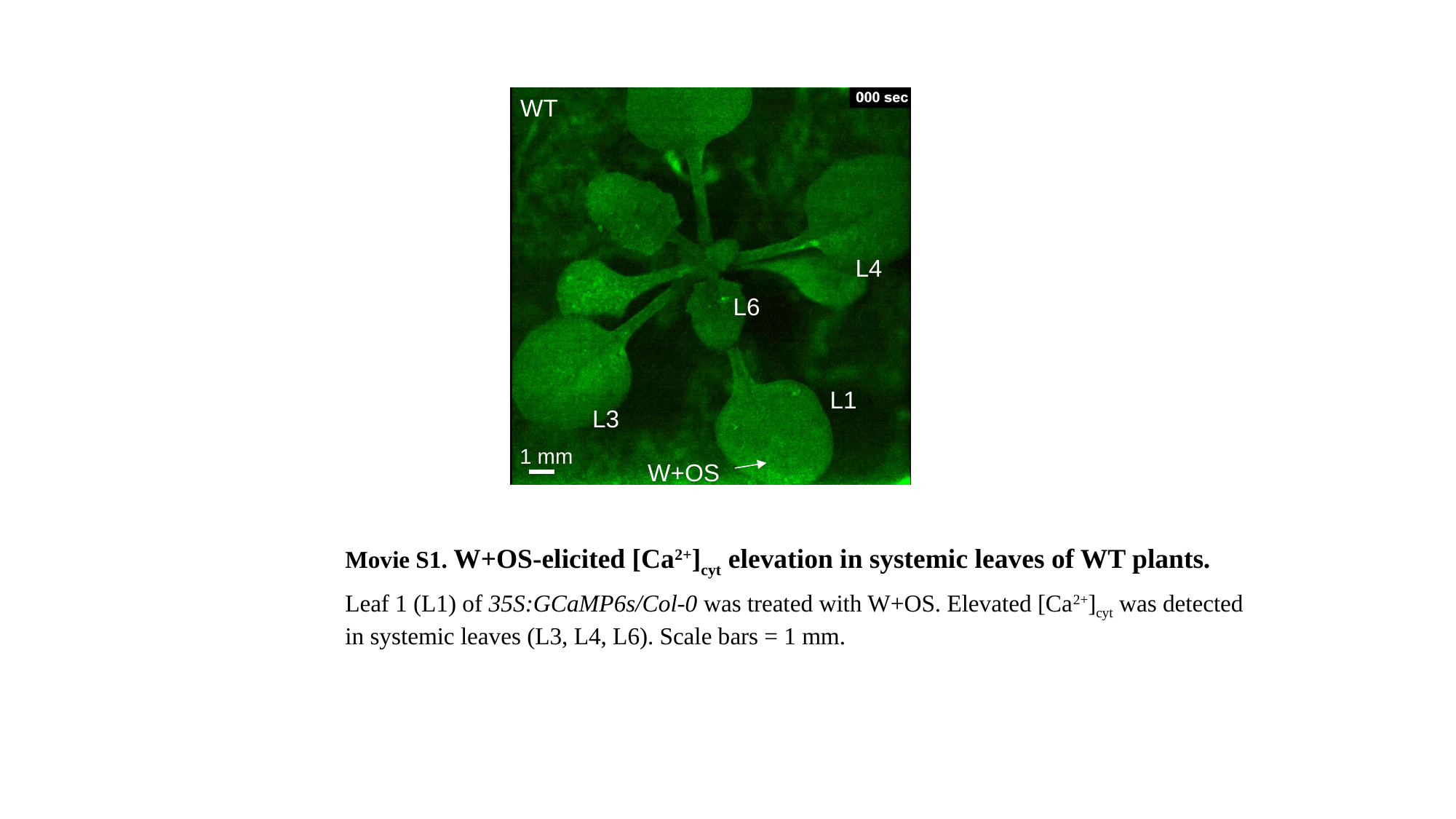

1 mm
WT
L4
L6
L1
L3
W+OS
Movie S1. W+OS-elicited [Ca2+]cyt elevation in systemic leaves of WT plants.
Leaf 1 (L1) of 35S:GCaMP6s/Col-0 was treated with W+OS. Elevated [Ca2+]cyt was detected in systemic leaves (L3, L4, L6). Scale bars = 1 mm.

## Slide 2
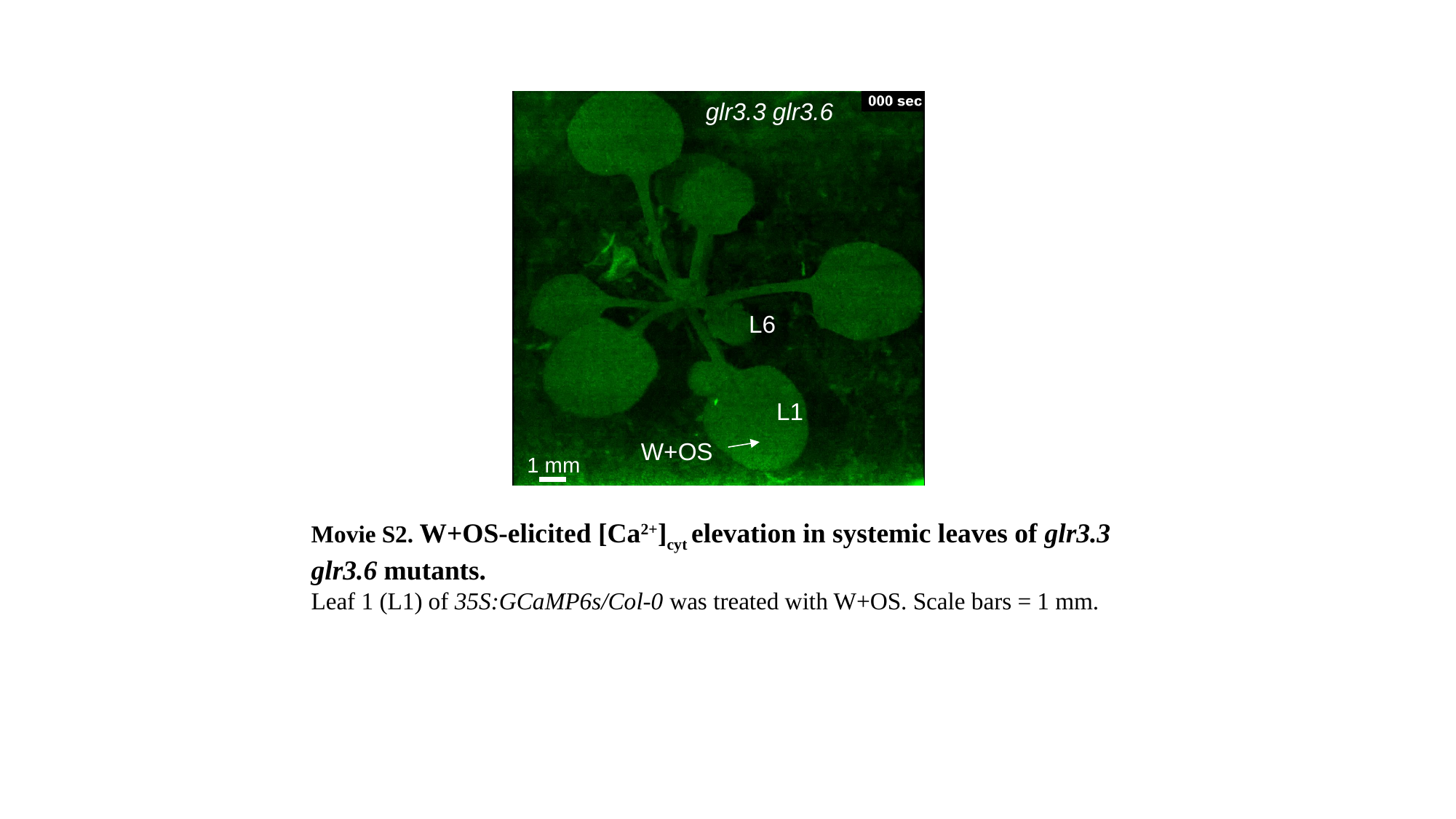

1 mm
1 mm
glr3.3 glr3.6
L6
L1
W+OS
W+OS
Movie S2. W+OS-elicited [Ca2+]cyt elevation in systemic leaves of glr3.3 glr3.6 mutants.
Leaf 1 (L1) of 35S:GCaMP6s/Col-0 was treated with W+OS. Scale bars = 1 mm.

## Slide 3
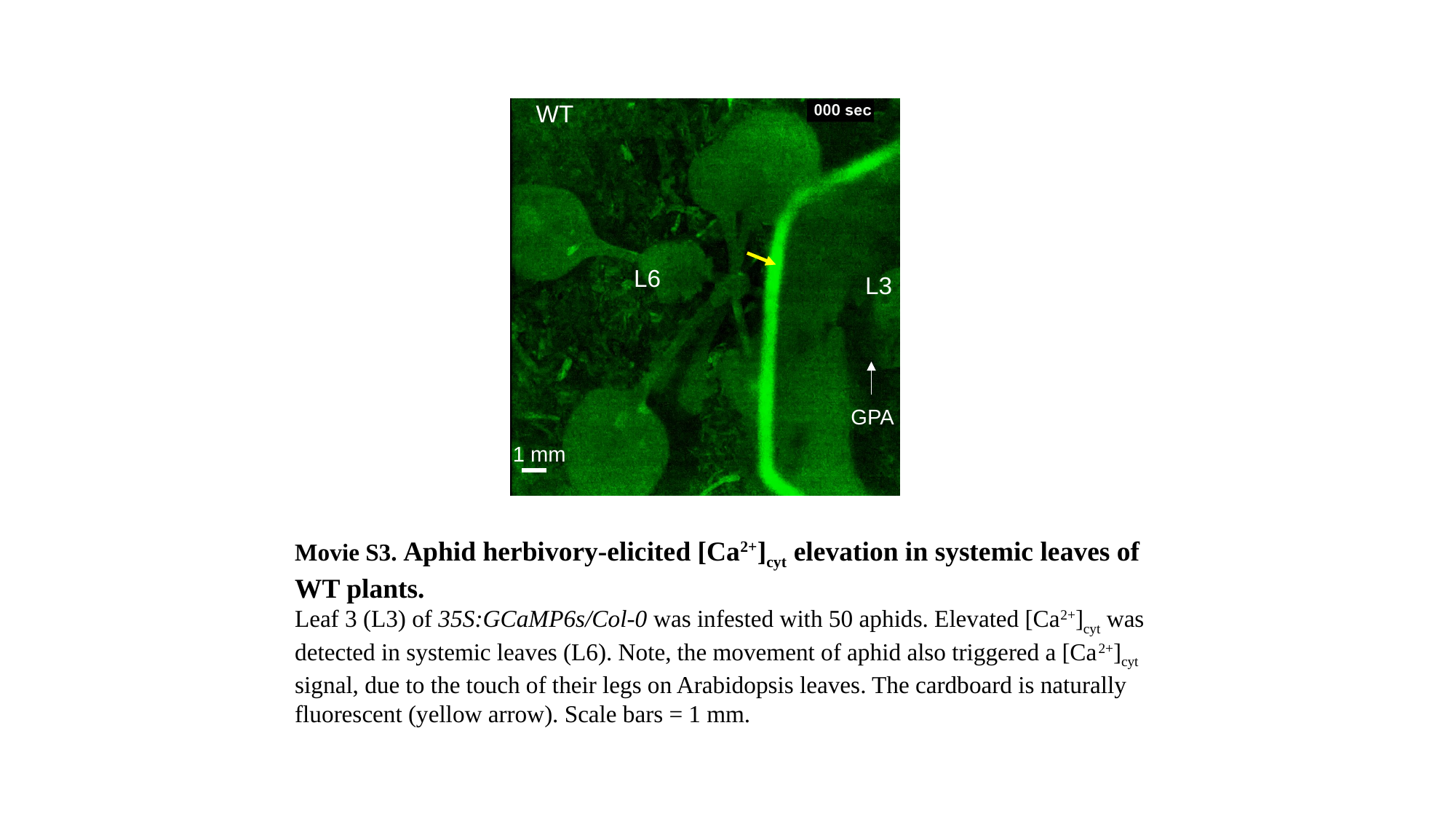

WT
1 mm
1 mm
L6
L3
GPA
Movie S3. Aphid herbivory-elicited [Ca2+]cyt elevation in systemic leaves of WT plants.
Leaf 3 (L3) of 35S:GCaMP6s/Col-0 was infested with 50 aphids. Elevated [Ca2+]cyt was detected in systemic leaves (L6). Note, the movement of aphid also triggered a [Ca2+]cyt signal, due to the touch of their legs on Arabidopsis leaves. The cardboard is naturally fluorescent (yellow arrow). Scale bars = 1 mm.

## Slide 4
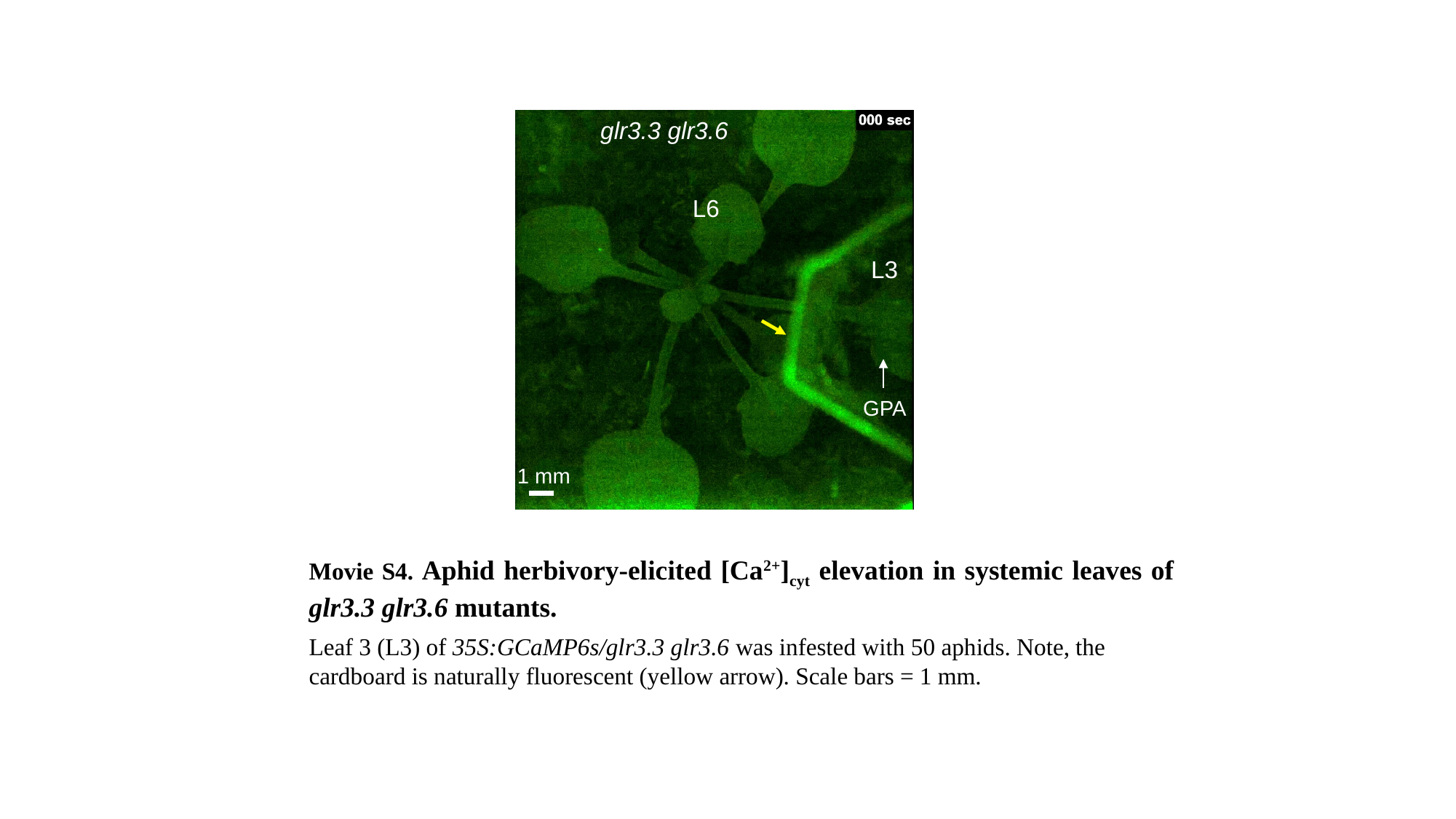

glr3.3 glr3.6
L6
L3
GPA
1 mm
Movie S4. Aphid herbivory-elicited [Ca2+]cyt elevation in systemic leaves of glr3.3 glr3.6 mutants.
Leaf 3 (L3) of 35S:GCaMP6s/glr3.3 glr3.6 was infested with 50 aphids. Note, the cardboard is naturally fluorescent (yellow arrow). Scale bars = 1 mm.
